# Towards a bridge between intracerebral and surface EEG signatures of conscious report

**DOI:** 10.1093/nc/niag011

**Published:** 2026-04-08

**Authors:** Silvana Lozito, Stefano Lasaponara, Jianghao Liu, Vincent Navarro, Katia Lehongre, Valerio Frazzini, Tal Seidel Malkinson, Fabrizio Doricchi, Paolo Bartolomeo

**Affiliations:** Department of Psychology, “Sapienza” University of Rome, Via dei Marsi 78, 00185 Rome, Italy; Sorbonne Université, Paris Brain Institute - ICM, Inserm, CNRS, AP-HP, Hôpital de la Pitié-Salpêtrière, 47 Bd de l'Hôpital, 75013 Paris, France; IRCCS Fondazione Santa Lucia, Via Ardeatina 306/354, 00179 Rome, Italy; PhD Programme in Behavioural Neuroscience, “Sapienza” University of Rome, Via dei Marsi 78, 00185 Rome, Italy; Department of Psychology, “Sapienza” University of Rome, Via dei Marsi 78, 00185 Rome, Italy; IRCCS Fondazione Santa Lucia, Via Ardeatina 306/354, 00179 Rome, Italy; Sorbonne Université, Paris Brain Institute - ICM, Inserm, CNRS, AP-HP, Hôpital de la Pitié-Salpêtrière, 47 Bd de l'Hôpital, 75013 Paris, France; Dassault Systèmes, 10 Rue Marcel Dassault, 78140 Vélizy-Villacoublay, France; Epilepsy Unit, AP-HP, Piti Salpêtrière Hospital, 47 Bd de l'Hôpital, 75013 Paris, France; CENIR - Centre de Neuro-Imagerie de Recherche, Paris Brain Institute, ICM, Hôpital de la Pitié-Salpêtrière, 47 Bd de l'Hôpital, 75013 Paris, France; Epilepsy Unit, AP-HP, Piti Salpêtrière Hospital, 47 Bd de l'Hôpital, 75013 Paris, France; Université de Lorraine, CNRS, IMoPA, 9 Avenue de la Forêt de Haye, 54000 Nancy, France; Department of Psychology, “Sapienza” University of Rome, Via dei Marsi 78, 00185 Rome, Italy; IRCCS Fondazione Santa Lucia, Via Ardeatina 306/354, 00179 Rome, Italy; Sorbonne Université, Paris Brain Institute - ICM, Inserm, CNRS, AP-HP, Hôpital de la Pitié-Salpêtrière, 47 Bd de l'Hôpital, 75013 Paris, France

**Keywords:** consciousness, EEG, ERPs, intracerebral electrodes, P300, attention

## Abstract

A recent study using intracerebral electroencephalography (EEG) recordings in human patients has documented the electrophysiological correlates of conscious reporting of near-threshold visual targets that followed supra-threshold peripheral spatial cues. Here, we aimed to bridge these intracerebral EEG events with corresponding surface recording, differentiating between conscious and nonconscious processing. We analysed the surface EEG of 10 patients from the intracerebral study. Due to a limited number of surface derivations, we pooled trials across participants to create a virtual participant for both surface and intracerebral analyses. Event-related potential (ERP) analysis revealed a significant positive deflection for Seen compared to Unseen targets in the 350–500 ms post-target window at frontal sites, consistent with a P3b component associated with conscious report. Time–frequency analysis revealed spectral dynamics associated with conscious report, including pretarget beta/gamma power modulations at frontal electrodes and post-target increased oscillatory activity at occipital sites. Trajectory *k*-means clustering of intracerebral data enabled us to identify two key patterns of post-target activity closely corresponding to the clusters from the original study: a Visual cluster exhibiting early (120–340 ms), transient responses, and an Accumulation cluster demonstrating gradual activity buildup (230–490 ms). Ridge regression analysis revealed that, compared to the Visual cluster, the Accumulation cluster contributed more to the prediction of report-related ERPs at the scalp level. These findings offer insights into bridging the gap between intracerebral recordings and surface EEG correlates of conscious report. They also highlight the greater contribution of late integrative mechanisms, compared to early sensory processes, in the conscious experience of behaviourally relevant targets.

## Teaser

- Intracerebral recordings identified Visual (occipito-temporal) and Accumulation (frontoparietal) neural patterns associated with conscious report.
- We aimed to bridge intracerebral EEG signatures with surface EEG correlates of conscious perception.
- Surface event-related potentials (ERPs) revealed a late positive deflection (350–500 ms) at frontal sites, consistent with P3b.
- Time-frequency analysis showed pre-target beta/gamma modulations and post-target oscillatory activity for seen vs unseen targets.
- Ridge regression revealed that the Accumulation cluster contributed more than the Visual cluster to surface ERP prediction.

## Introduction

One of the greatest challenges in neuroscience is understanding the neural correlates of conscious perception (Martín-Signes et al. 2024). Recent EEG surface-level studies have identified several electrophysiological signatures that consistently differentiate conscious from nonconscious states across diverse contexts and sensory modalities. Among these, the P3b event-related potential (ERP) has been proposed as a key neural signature of conscious access. For example, Naccache (2018) interprets this positive, slow potential shift as reflecting net inhibition across broad neural networks, selectively reducing activity in nonrelevant assemblies to focus resources on consciously accessed content within the Global Neuronal Workspace (GNW) theory (Dehaene 2001). The consistent observation of P3b across sensory modalities and types of conscious content suggests it may serve as a generalizable marker of conscious access, supporting the GNW theory’s role in enabling conscious perception by making information widely accessible. Another proposed ERP marker of phenomenal consciousness is the Visual Awareness Negativity (VAN), which appears earlier than P3b, around 200 ms poststimulus, in posterior sensory regions (Koch et al. 2016). This early component has been generalized within the Perceptual Awareness Negativity (PAN) framework, which encompasses analogous awareness negativities across sensory modalities (Dembski et al. 2021). While these EEG markers reveal modality-specific and temporally distinct neural signatures of consciousness across distributed sensory cortices, the GNW theory proposes that the prefrontal cortex (PFC) serves as a crucial hub that integrates and broadcasts sensory information through extensive cortical interactions, thereby underpinning conscious awareness (Dehaene 2001). Electrophysiological studies suggest that the PFC acts through synchronized beta-band activity with posterior areas, facilitating the dynamic update and stability of conscious content across cortical networks (Panagiotaropoulos 2024). Furthermore, the PFC involvement as a potential generator of the VAN and P3b components emphasizes its centrality in modulating conscious states across sensory modalities. In particular, the phenomenon of ‘ignition’—a sharp, nonlinear increase in neural activity within the PFC—has been shown to facilitate access and integration within the workspace, where it interacts with temporal and parietal regions to sustain reportable conscious perception (Del Cul et al. 2007, Dehaene and Changeux 2011).

The use of intracerebral recordings in humans has recently advanced our understanding of the electrophysiological correlates of conscious report. Liu et al. (2023) investigated intracerebral neural activity in patients with drug-resistant epilepsy who had been implanted with intracerebral electrodes. During the recordings, patients were presented with near-threshold peripheral Gabor patches, each preceded by nonpredictive peripheral attentional cues. They were asked to discriminate the orientation of the Gabor patches and report whether or not they perceived them. A trajectory *k*-means clustering analysis (Seidel Malkinson et al. 2024) revealed three main neural patterns associated with conscious report and its interaction with attention. First, an early transient post-target activity appeared in bilateral occipito-temporal areas (referred to as the ‘Visual cluster’), followed by sustained activity in right-hemisphere frontoparietal regions linked by the second and third branches of the superior longitudinal fasciculus (SLF II–III). This activity was enhanced for cued trials and accompanied conscious reports (‘Sustained cluster’). Subsequently, a late accumulation of activity (>300 ms post-target) emerged in frontoparietal circuits connected by SLF I–III (‘Late Accumulation cluster’). Second, a right-hemispheric cluster corresponding to the ventral attention network (SLF III) (Corbetta and Shulman 2002), referred to as the ‘Reorienting cluster’, showed increased activity for reported uncued targets. Third, activity in the left dorsolateral prefrontal cortex—independent of cueing—was also associated with conscious target detection. These findings suggest that conscious report depends on distinct, hemisphere-asymmetric frontoparietal networks (Bartolomeo and Seidel Malkinson 2019, Bartolomeo et al. 2025). Notably, these results support and further specify the role of attentional ignition in the conscious perception of near-threshold stimuli, as predicted by the GNW theory.

The present study aimed to bridge the gap between invasive and noninvasive neurophysiological signatures of conscious report. We analysed surface EEG data collected simultaneously with intracerebral recordings during the experiments performed by Liu et al. (2023) to determine which neural signatures of consciousness identified at the intracerebral level are detectable at the scalp level and how different neural processes contribute to these surface manifestations. This approach offers an opportunity to examine how neural processes identified through precise intracerebral measurements manifest in the more clinically accessible but spatially less accurate signals captured by surface EEG. The study by Liu et al. (2023) provides a testable foundation for such a comparison, having already established the intracerebral dynamics of conscious perception through a comprehensive analysis of spatiotemporal patterns. Here, we investigated the correspondence between intracerebral indices of the seen/unseen distinction and their surface EEG correlates. We employed a multi-level analytical approach to conduct a systematic investigation: First, we used ERP and time–frequency analyses to identify consciousness-related signals in surface EEG. Second, we replicated the trajectory *k*-means clustering analysis originally employed by Liu et al. (2023) on the subset of intracerebral contacts included in the present study. This allowed us to identify the key neural patterns associated with conscious report. Finally, we employed ridge regression with bootstrap validation to quantify the contribution of these intracerebral patterns to the observed surface EEG signals. However, the limited surface electrode coverage—primarily frontopolar and frontotemporal sites—precluded a full characterization of all classical awareness–related signals. Despite this limitation, our approach enabled us to trace intracerebrally recorded consciousness-related activity to its surface expressions, clarifying which neural mechanisms of consciousness—particularly those reflected in late ERP components and prestimulus oscillatory activity—are accessible through noninvasive recordings.

## Materials and Methods

### Participants’ surface and intracerebral EEG recordings

Our initial sample consisted of 13 patients who underwent presurgical evaluation of pharmaco-resistant focal epilepsy, as reported in the study by Liu et al. (2023). However, one of the patients did not have surface EEG available, and two others were excluded during the preprocessing stage due to excessive noise in the data, likely affected by ocular or muscular artefacts. Thus, our final sample included 10 patients (mean age ± SD: 34 ± 8.7; 7 women; 9 right-handed).

All participants had normal or corrected-to-normal vision and provided written informed consent, with approval from the local ethics committee of the Department of Neurosurgery at Hôpital Pitié-Salpêtrière, Paris, France (CPP Paris VI, Pitié-Salpêtrière Hospital, INSERM C11–16 (2012–20); CPP INSERM C19–55). Patients differed in their prescribed medical treatments, which were reduced or discontinued after implantation, depending on each patient’s medical needs and the delay between surgery and experimental recording (range, 3–14 days). Patients were implanted with 4–13 multi-lead stereotactic depth electrodes (AdTech®, Wisconsin), each comprising 4–12 platinum contacts (1.12 mm in diameter and 2.41 mm in length) with nickel–chromium wiring. The distance between the centres of two contiguous contacts was 5 mm. The location of electrode implantation was based exclusively on clinical criteria. In two patients, neural activity was recorded using a 128-channel clinical video-EEG system (SD LTM 64 BS, Micromed® S.p.A., Italy), sampled at 1024 Hz with a band-pass filter of 0.15–250 Hz. In the other eight patients, the recording was implemented with a Neuralynx system (ATLAS, Neuralynx®, Inc., Bozeman, MO), which allowed recording up to 160 depth-EEG channels sampled at 4 kHz with a 0.1–1000 Hz band-pass. Both systems recorded intracerebral and surface EEG simultaneously. The patient-dependent least active contact, preferably in the white matter, was selected as the reference electrode for intracerebral recordings, while M1–M2 electrodes (placed on the earlobes) served as a reference for surface EEG. Surface electrode positions varied among patients due to clinical constraints related to the implantation sites.

### Intracerebral EEG preprocessing

Spatial localization of each electrode was performed using the Epiloc toolbox (https://icm-institute.org/en/cenir-stim), which coregistered pre-implantation MRI scans (1.5T or 3T) with postimplantation CT and MRI scans. Pre-implantation MRI, postimplantation MRI, and postimplantation CT were normalized to Montreal Neurological Institute (MNI) space. Contact localization was automatically labeled according to the Desikan–Killiany–Tourville atlas parcellation using the Freesurfer image analysis suite (https://surfer.nmr.mgh.harvard.edu/) embedded in Epiloc, followed by manual verification and correction when necessary. Signal preprocessing was performed using MATLAB (R2023b, The MathWorks, Inc.) and the FieldTrip toolbox. All signals were downsampled to 512 Hz. A bipolar montage was applied, in which each contact was re-referenced to its adjacent neighbour on the same electrode, ensuring that the recorded signals originated from a cortical volume centered between the two contacts. Bipolar contact coordinates were computed as the mean MNI coordinates of the two adjacent contacts composing the bipole. An initial visual inspection of continuous signals was conducted to remove time segments containing transient epileptic or interictal activity, and contacts showing excessive epileptic spikes or located near suspected epileptic foci were excluded. Trial epochs were extracted from −1300 to 1200 ms relative to the target onset. A second artifact rejection was performed on the epoched data, excluding trials and contacts with excessive maximal signal, *z*-values, variance, or kurtosis of the signal distribution. After signal preprocessing, 727 bipolar contacts out of 887 were retained. Due to the exclusion of three participants in the present study (see the Participants’ Surface and Intracerebral EEG Recordings section), the corresponding intracerebral contacts were also excluded, resulting in a final sample of 541 contacts from 10 patients for all subsequent analyses. A pseudo-whole-brain analysis approach was adopted by pooling contacts across patients on a standardized brain in MNI space. High-frequency broadband (HFBB) power (70–140 Hz), considered a proxy of local neuronal population spiking activity, was extracted from each bipolar contact using wavelet time–frequency transformation with Morlet wavelets (ft_freqanalysis in FieldTrip) across fourteen equally spaced center frequency bands. HFBB power time courses were retained from −800 to 1000 ms relative to target onset to avoid edge artifacts related to the 1/*f* signal drop-off. Baseline normalization was applied trial-by-trial using *z*-scores relative to the 200 ms period prior to cue onset. Finally, HFBB power was downsampled to 100 Hz for further analyses.

### Surface EEG preprocessing

**Figure 1:**
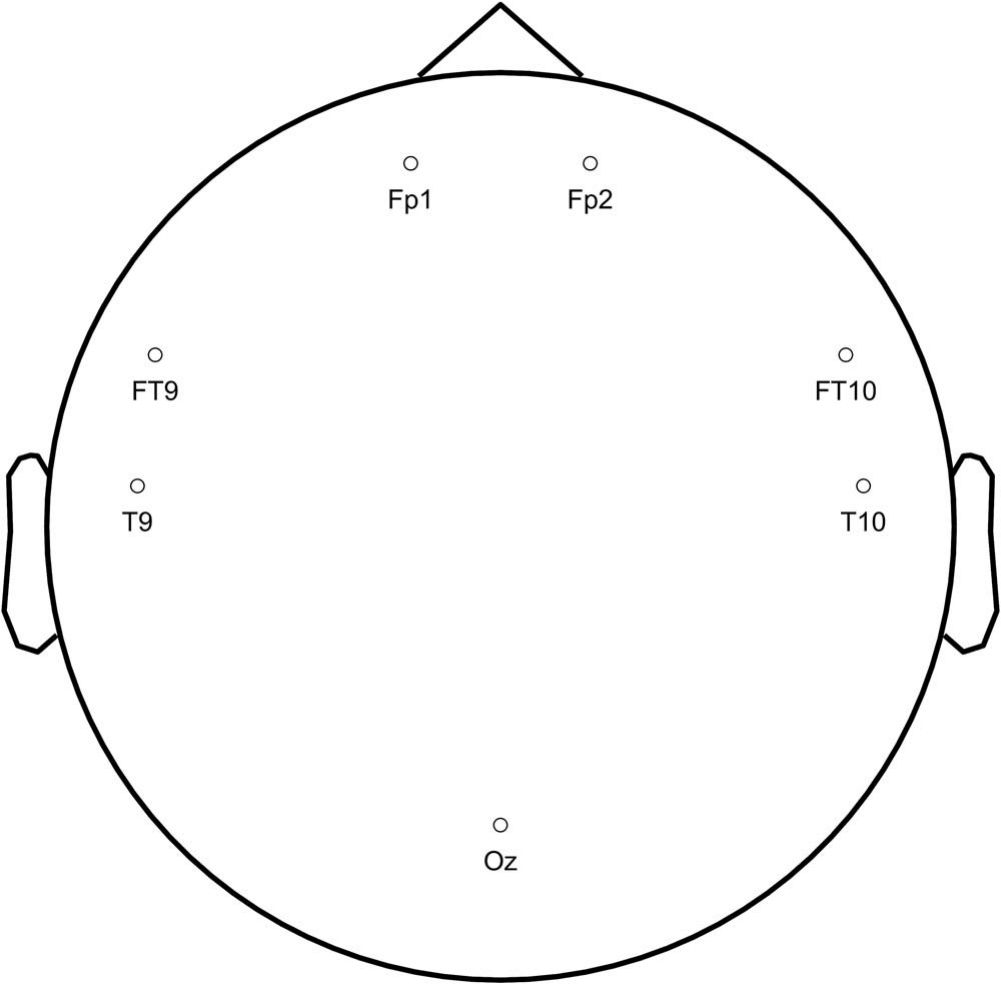
Electrode placement for surface EEG recordings.

Surface EEG data were recorded at different positions across patients. For analysis, we included only electrodes positioned at Fp1, Fp2, FT9, FT10, Oz, T9, and T10 sites (Fig. 1), as other positions lacked sufficient coverage across the patient sample. Surface electrodes were positioned at a distance from the skull entry points of the intracranial electrodes (Lehongre et al. 2022). For microbiological safety reasons, no additional scalp EEG electrodes could be added. Specifically, data from electrodes Fp1, Fp2, FT9, and FT10 were recorded from 10 patients, data from Oz were available for 7 patients, and data from T9 and T10 were recorded from 6 patients (see Table 1 for the detailed distributions of the electrodes across participants).

**Table 1:** Electrode positions used for surface EEG recordings in each patient

**Patient ID**	**Electrode positions**
P7	Fp1, Fp2, FT9, FT10, T9, T10
P9	Fp1, Fp2, FT9, FT10
P12	Fp1, Fp2, FT9, FT10
P15	Fp1, Fp2, FT9, FT10, Oz
P19	Fp1, Fp2, FT9, FT10, Oz
P21	Fp1, Fp2, FT9, FT10, Oz, T9, T10
P22	Fp1, Fp2, FT9, FT10, Oz, T9, T10
P24	Fp1, Fp2, FT9, FT10, Oz, T9, T10
P28	Fp1, Fp2, FT9, FT10, Oz, T9, T10
P30	Fp1, Fp2, FT9, FT10, Oz, T9, T10

Signal preprocessing was conducted using MATLAB (R2023b, The MathWorks, Inc.) and the FieldTrip toolbox. First, all signals were downsampled to 512 Hz. Independent component analysis (ICA) was then applied to remove electrooculography (EOG) artefacts using the ‘runica’ method, yielding as many components as the number of available channels (four to seven per participant). Each component was visually inspected by examining both its topographical distribution (ft_topoplotIC) and its time course across trials (ft_databrowser in ‘component’ viewmode). Components showing patterns consistent with ocular artifacts (e.g., frontal distribution and characteristic blink-related time course) were identified and removed; typically, one component was removed per participant. After the ICA, a visual inspection of the remaining data was performed using FieldTrip’s summary mode (ft_rejectvisual), which allows identification of outlier trials based on statistical metrics such as mean, variance, and kurtosis. Trials identified as outliers were excluded from subsequent analyses. Finally, for the ERP analysis, a low-pass filter with a cutoff frequency of 30 Hz was applied to remove high-frequency noise. This filter was not applied to the time–frequency analysis.

### Experimental task

**Figure 2:**
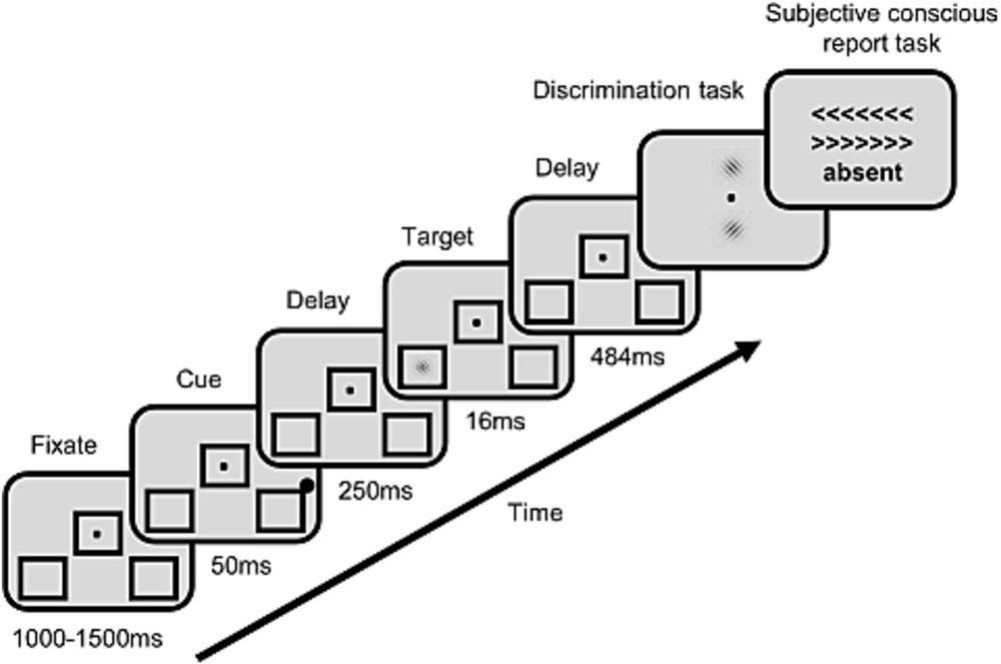
Near-threshold target detection task. After a fixation dot lasting 1000–1500 ms, a peripheral non-predictive dot cue occurred for 50 ms, followed by a left-sided or right-sided near-threshold tilted Gabor target presented for 16 ms. After a delay of 484 ms, participants discriminated the direction of tilt and reported whether the Gabor was present or not. In 20% of trials (catch trials), the Gabor target was absent. Individual contrasts based on an individual calibration procedure were used across all conditions.

As described in Liu et al. (2023), participants performed a visual detection and discrimination task (see Fig. 2). Stimuli were presented using E-Prime 2.0 on a laptop with a 60 Hz refresh rate. Trials began with a fixation point and three placeholder boxes. After a variable delay (1000–1500 ms), a brief (50 ms) cue (a low-contrast black dot) appeared near one of two peripheral boxes in the lower visual field. Following a 300 ms interval, a target stimulus (a tilted Gabor patch, 16 ms duration) was presented in one of the peripheral boxes (or omitted in catch trials). Participants then performed two forced-choice tasks: (i) discriminating the tilt of the Gabor patch (if present) and (ii) reporting whether they perceived the Gabor patch at all (subjective awareness). The cue was nonpredictive of the target location (50% valid, 50% invalid). Each participant completed eight blocks of 110 trials (88 target-present, 22 catch). The total number of trials effectively recorded varied across participants. Before the recording blocks, participants underwent a target contrast calibration session to estimate their individual perceptual threshold contrast for 50% of the seen targets. The contrast level of the presented target was individually calibrated for each patient, with a typical contrast range of 10%–25%. Patients were asked about their subjective perception of the cue and confirmed that it was easily perceived and consistently visible throughout the experiment.

## Statistical analyses

### Surface EEG analysis

#### Event-related potentials

ERPs were analyzed to investigate neural responses associated with conscious and unconscious perception. As in the case of intracerebral recording (Liu et al. 2023), ERPs were analysed within a time window of −500 to +500 ms relative to the onset of the target stimulus. Baseline correction was performed using a 200 ms window preceding the cue onset (−500 to −300 ms relative to the target onset). Consistent with the original study (Liu et al. 2023), only trials with correct orientation discrimination for Seen targets were included, as incorrect responses might have been the consequence of unreliable perception of the target. In contrast, all trials for Unseen targets were included, regardless of orientation discrimination accuracy, since any correct response in this condition would likely reflect random guessing rather than genuine conscious processing. Due to the limited sample size and variability in electrode availability across participants, trials from all participants were pooled together to create a virtual participant, with trials rather than individual participants as the unit of analysis. Before preprocessing, pooling across participants yielded a total of 3103 target-present trials for electrodes Fp1, Fp2, FT9, and FT10; 2 191 for Oz; and 1823 for T9 and T10. To assess differences between the ERP waveforms for the Seen and Unseen conditions, we applied a one-dimensional (1D) cluster-based permutation test (Cohen 2014, Pérez et al. 2021). Differences between conditions were computed at each time point using independent-samples *t*-tests, and contiguous time points with *P* < .05 were grouped into clusters. The cluster-level statistic was computed as the sum of *t*-values within each cluster. To evaluate cluster significance, a null distribution was generated by performing 1000 permutations of the condition labels. A cluster was deemed statistically significant if its summed *t*-value exceeded the 95th percentile of the null distribution (*P* < .05). In addition, we examined potential differences as a function of the target’s lateral position relative to the hemisphere in which each electrode was located (ipsilateral or contralateral). Similarly, significant clusters were identified using *t*-tests combined with cluster-based permutation statistics (1D cluster correction, *P* < .05). To assess potential interaction effects, a two-way analysis of variance (ANOVA) with an unbalanced design was conducted to account for the varying trial counts across the conditions. The two factors included were Validity (Valid vs. Invalid) and Consciousness (Seen vs. Unseen). To further investigate the interaction between the lateralized nature of the ERP components and the consciousness effect, we conducted a two-way ANOVA with an unbalanced design with Consciousness (Seen vs. Unseen) and Electrode Laterality (Ipsilateral vs. Contralateral) as factors. For each ANOVA, we applied an false discovery rate (FDR) correction to account for multiple comparisons. Finally, we employed a Linear Mixed-Effects (LME) model on ERPs recorded via surface EEG to also account for variability across trials and participants (see Supplementary Material, Section S1 for details).

#### Time–frequency

To explore the oscillatory dynamics underlying conscious and unconscious visual processing, we conducted a comprehensive time-frequency analysis using wavelet transformation. This method allowed us to capture changes in neural oscillatory power across a broad range of frequencies, offering insights into both the temporal and spectral characteristics of brain responses to stimuli in Seen and Unseen conditions. The time–frequency analysis was performed using the FieldTrip toolbox implemented in MATLAB (R2023b, The MathWorks, Inc.). For each participant, we processed the surface EEG data across a frequency range of 5–50 Hz, covering key theta, alpha, beta, and gamma frequency bands. The wavelet width was set to adapt based on the frequency of interest (Spadone et al. 2021), starting from seven cycles at lower frequencies and increasing proportionally for higher frequencies:


$$ width=7+\frac{frequency-10}{4} $$

The time window of analysis extended from −800 to 500 ms, with 0 ms corresponding to the target onset. Prior to the time–frequency analysis, a baseline correction (500 ms precue interval) was applied to normalize the power values. The time–frequency data were computed for each electrode separately, pooling together the trials from all participants to create a virtual participant, similar to the approach employed in the ERP analysis. The resulting power spectra were stored for both the Seen and Unseen targets for all the trials of the virtual participant. To determine the statistical significance of oscillatory power between Seen and Unseen targets, we applied a cluster-based permutation analysis controlling for multiple comparisons across both time and frequency dimensions. For each electrode, we employed an independent *t*-test with a Monte Carlo method to compare the two conditions (Seen and Unseen), with 1000 permutations. The statistical analysis focused on detecting both positive and negative clusters—regions where either Seen > Unseen or Unseen > Seen—with an α level of 0.05. Significant clusters were identified based on the summed *t*-values of adjacent time–frequency points exceeding a predefined threshold. This cluster-based approach increases statistical sensitivity by aggregating effects across neighbouring data points, thereby making it more robust for identifying meaningful differences in oscillatory activity between conditions.

### Intracerebral EEG analysis: trajectory *k*-means clustering

HFBB power is considered a proxy of the spiking activity of the local neuronal ensemble. Here, to ensure methodological consistency with the surface EEG (ERP) analysis, we reanalysed the HFBB signals from the original dataset by Liu et al. (2023). Specifically, given the exclusion of 3 participants at the surface level, we performed the clustering analysis on the final sample of 10 participants. We applied a *k*-means-based clustering approach developed by Seidel Malkinson et al. (2024) to classify contacts by their temporal profiles, implemented in MATLAB (R2023b, The MathWorks, Inc.). This data-driven approach captured the prototypical patterns of neural dynamics that might be sensitive to cue validity and Seen/Unseen reports. We conducted clustering on all bipolar contacts. In each contact, we took the trajectories of the mean target-locked activity across an eight-dimensional condition space (target side, cue validity, and Seen/Unseen report). Activity across conditions was *z*-scored relative to the distribution of the trials’ entire duration. Contacts were then iteratively partitioned (10 000 iterations) into 2–12 clusters, in which each contact was assigned to the cluster with the nearest centroid trajectory. This was achieved by minimizing the sum of time-point-by-time-point Manhattan distances across conditions, to quantify trajectory similarity while preserving temporal order. Based on silhouette evaluation (silhouette in Matlab), we identified the optimal cluster solution, which yielded the highest average silhouette score.

### Relationship between surface EEG and intracerebral recording: ridge regression

Here, using ridge regression (see Supplementary Material, Section 3 for details on the mathematical framework, optimization procedure, *k*-fold cross-validation approach, and bootstrap validation method), we investigated the relationship between intracerebral activity and observed surface-level dynamics. To this end, after identifying clusters consistent with the original study (Liu et al. 2023), we evaluated their roles in predicting surface-level ERP signals associated with conscious perception. To ensure compatibility between the two datasets, the surface EEG signals were downsampled to 100 Hz, aligning their sampling rate with that of the HFBB signals extracted from the intracerebral data. Specifically, we computed the ERP differential signal between the two conditions for each analysed surface electrode that showed significant differences between trials with seen and unseen targets. The same differential signals were computed for the HFBBs at the intracerebral level. These differential signals reflect neural dynamics associated with conscious report and were averaged across trials from participants whose intracerebral contacts were included in at least one cluster identified by *k*-means clustering. However, T9 and T10 electrodes were excluded from the analysis because not all participants contributing to the clusters had these electrodes available in their surface EEG setup, and no significant conscious report effect was observed at these locations on the surface level. Similarly, Oz was excluded because no significant effect of conscious report was observed in the ERP analysis. Intracerebral predictors and the surface ERP signals were normalized by subtracting their respective means and dividing by their standard deviations. The analysis focused on the 500 ms interval following target onset, as this timeframe was identified in our ERP study as critical for capturing consciousness-related effects. The prestimulus window was excluded from the regression analysis because no consciousness-related ERP differences were observed in that interval, and, consistent with the original study (Liu et al. 2023), no significant modulations in intracranial HFBB data were observed during the cue-to-target period.

## Results

### Surface EEG results

#### Event-related potentials

**Figure 3:**
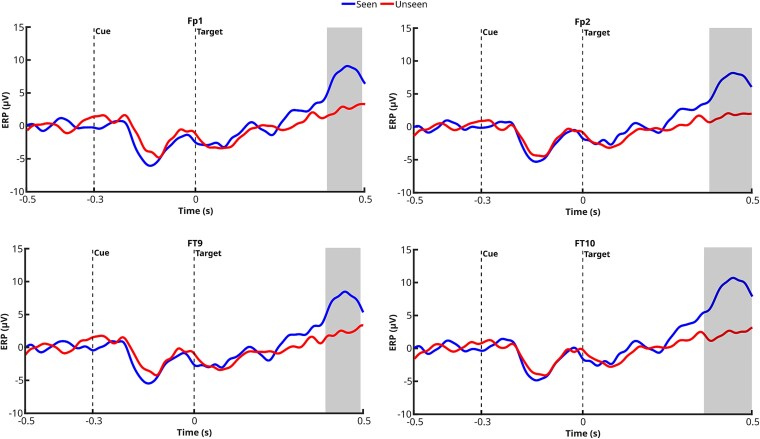
Comparison of ERP waveforms between the Seen and Unseen conditions across electrodes. The trial counts for Fp1, Fp2, FT9, and FT10 were 1128 (Seen) and 1241 (Unseen). Grey-shaded areas indicate significant difference clusters, identified through one-dimensional cluster-based statistics corrected for multiple comparisons (*P* < .05).

After preprocessing, a total of 2369 trials (1128 Seen, 1241 Unseen) were included in the analysis for electrodes Fp1, Fp2, FT9, and FT10; 1 670 trials (802 Seen, 868 Unseen) for Oz; and 1351 trials (637 Seen, 714 Unseen) for T9 and T10. Significant clusters for electrodes Fp1, Fp2, FT9, and FT10 (see Fig. 3) were observed in the post-target onset interval, with clusters extending between ~360 and 500 ms poststimulus onset, depending on the electrode. As for electrodes T10 and Oz, initial *t*-tests suggested potential differences between Seen and Unseen targets; however, these effects did not survive the cluster-based permutation correction. No significant differences between Seen and Unseen targets were obtained for T9, even before clustering. The lack of significant differences at electrodes T10, Oz, and T9 does not necessarily imply the absence of condition-related effects. Instead, these effects were likely less robust due to the limited number of participants, which reduced statistical power.

**Figure 4:**
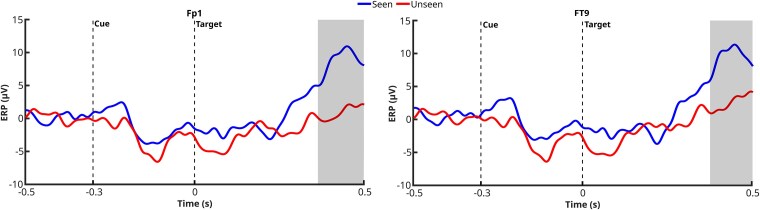
ERP waveforms for Seen and Unseen targets at Fp1 and FT9, for contralateral targets. Gray-shaded areas represent significant clusters identified using *t*-tests and cluster-based permutation statistics (1D cluster correction, *P* < .05). Trial counts were Seen = 558, Unseen = 628.

**Figure 5:**
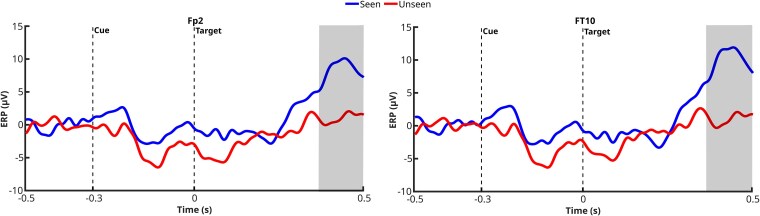
ERP waveforms for Seen and Unseen targets at Fp2 and FT10, for ipsilateral targets. Grey-shaded areas represent significant clusters identified using *t*-tests and cluster-based permutation statistics (1D cluster correction, *P* < .05). Trial counts were Seen = 558, Unseen = 628.

The same analysis, conducted while accounting for laterality, revealed a consistent lateralization pattern across the significant clusters identified. For electrodes in the left hemisphere (Fp1 and FT9), significant clusters associated with the conscious condition (‘Seen’ > ‘Unseen’) emerged in the contralateral trials (i.e. when the target appeared on the right side, 558 Seen, 628 Unseen) (see Fig. 4). Conversely, for electrodes in the right hemisphere (Fp2 and FT10), significant clusters were found in the ipsilateral trials (i.e. when the target appeared on the same side as the electrode, right side, 558 Seen, 628 Unseen) (see Fig. 5). This pattern suggests a lateralized modulation of the ERP effects associated with conscious processing, potentially reflecting differential contributions from interhemispheric communication or attentional mechanisms.

The two-way ANOVA with Validity and Consciousness as factors revealed no significant interactions lasting at least 30 ms, even before correction for multiple comparisons. In contrast, the two-way ANOVA with Consciousness and Laterality as factors, after FDR correction, revealed a significant interaction between Consciousness and Laterality. These effects were localized to FT9 during the post-target period (410–500 ms) and to FT10 during the cue period (−105 to −54 ms).

#### Time–frequency results

**Figure 6:**
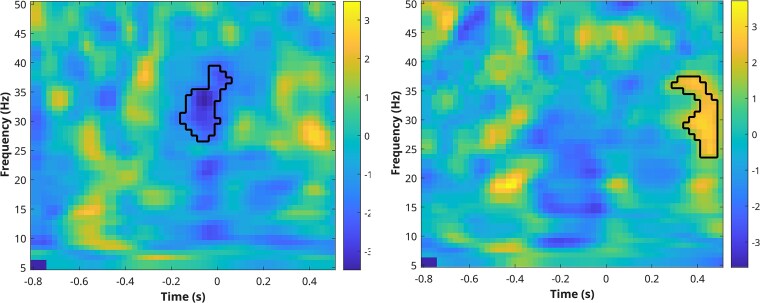
Left: Fp1. Significant cluster (Unseen > Seen, cluster-based permutation test, *P* < .05) outlined in black countour. Right: Oz. Significant cluster (Seen > Unseen, cluster-based permutation test, *P* < .05) outlined in black countour. The color bar represents *t*-values.

Differences in oscillatory power between Seen and Unseen targets’ trials were observed for the Fp1 electrode. Specifically, we observed a significant increase in beta and gamma band activity (27–40 Hz) for Unseen targets compared to Seen ones. This effect was prominent in the 200 ms preceding target onset (see Fig. 6, left).

At the Oz electrode, a late increase in beta- and gamma-band power for Seen versus Unseen conditions was detected, with the effect observed after 300 ms from target onset (see Fig. 6, right).

### Intracerebral recording results

#### Trajectory *k*-means clustering

**Figure 7:**
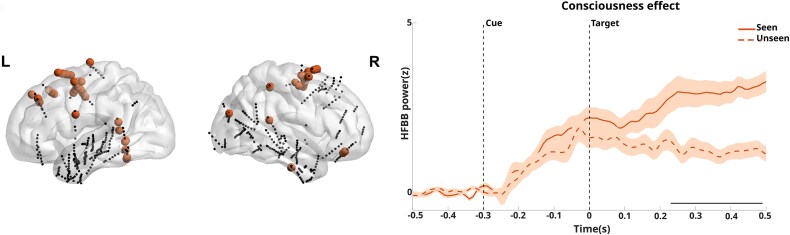
Left: Cluster contacts localization (red dots) for Late Accumulation pattern. Right: Comparison of target locked neural activity for seen and unseen trials. Black horizontal bar for all *P*s < .05, Holm–Bonferroni corrected, red shading is ±SEM across electrodes.

**Figure 8:**
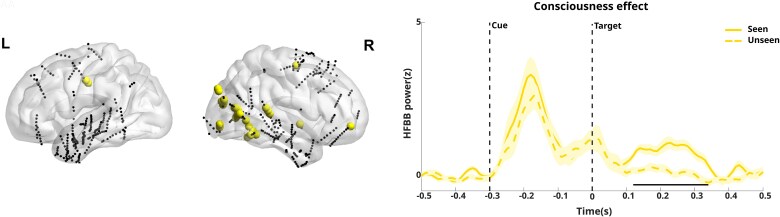
Left: Cluster contacts localization for the Visual cluster (yellow dots). Right: Comparison of target locked neural activity for seen and unseen trials. Black horizontal bar for all *P*s < .05, Holm–Bonferroni corrected, yellow shading is ±SEM across electrodes.

One of the primary differences between our data and the original study lies in the number of intracerebral electrodes analysed. Liu et al. (2023) utilized 727 intracerebral contacts pooled from all the participants. However, because three patients were excluded from our surface EEG dataset, we also excluded the corresponding intracerebral electrodes for these participants, reducing the number of contacts from 727 to 541. *K* = 10 was identified as the optimal clustering solution (see Fig. S1 in the Supplementary Material, Section 2, for details). For each cluster, we computed the proportion of cluster contacts attributed to each patient (see Fig. S2, Supplementary Material, Section 2, for details). The clustering analysis revealed two primary patterns that align closely with two of the five patterns identified in the original intracerebral study (Liu et al. 2023) (see Fig. S3, Supplementary Material, Section 2, for details), namely, the Late accumulation cluster and the Visual patterns. The late accumulation pattern is associated with the gradual ramping up of neural activity over time, potentially linked to integrated processing for conscious perception, whereas the visual pattern reflects stimulus-driven visual responses, consistent with early sensory processing. For each of the two clusters, the neural dynamics were compared between Seen and Unseen targets’ trials, allowing us to assess the presence of the consciousness effect in each pattern to confirm the findings observed in Liu et al. (2023). Figures 7 and 8 show the distribution of electrodes contributing to each cluster and the comparison of target-locked neural activity for Seen and Unseen targets’ trials, respectively, for the Late Accumulation and Visual clusters, replicating previously reported results (Liu et al. 2023).

A significant consciousness effect was observed in both patterns ([0.23 0.49] for the accumulation cluster and [0.12 0.34] for the visual cluster). This analysis highlights that both late accumulation and visual clusters demonstrate robust neural differences between trials of Seen and Unseen targets.

In addition, the clustering analysis showed significant effects for the interaction [i.e. (Seen Valid-Seen Invalid) − (Unseen Valid-Unseen Invalid)] between conditions. However, no significant effects lasting longer than 30 ms were observed.

### Relationship between surface EEG and intracerebral recording

#### Ridge regression

**Figure 9:**
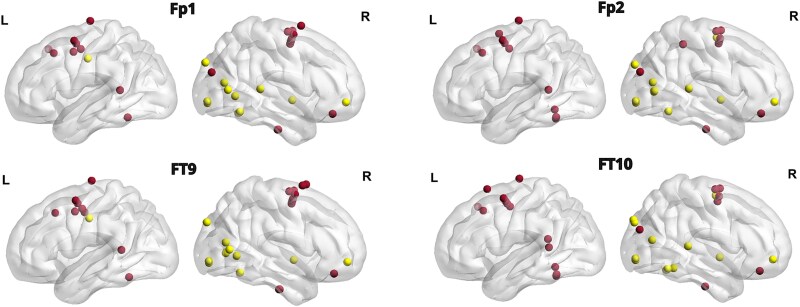
Localization of reliable predictors for Visual cluster (yellow dots) and Late accumulation cluster (red dots) for Fp1 (top left), Fp2 (top right), FT9 (bottom left), and FT10 (bottom right).

Cross-validation analysis revealed a consistent pattern in the mean squared error (MSE) across the range of log(λ) values for each electrode. While noticeable variability is observed for larger values of log(λ), the MSE remains stable around the optimal λ values identified through the procedure (see Fig. S4, Supplementary Material, Section 3**,** for details). This consistency reinforces the robustness of the model’s performance in identifying reliable regularization parameters, despite variations in the broader range of λ values. Notably, for some electrodes, the optimal λ values were consistent across several random seeds, further supporting the stability of the identified regularization parameters. Using a bootstrapping approach, we identified reliable predictors as those whose 95% confidence intervals did not include zero across all seeds. These predictors were considered robust, as their contribution was consistent across different partitions of the dataset and unlikely to be due to chance. The anatomical localization of reliable predictors for each cluster and surface electrode is reported in Table S1 (Supplementary Material) and illustrated in Fig. 9. This distribution revealed distinct patterns for each surface electrode. For Fp1, reliable Visual cluster intracerebral predictors were primarily located in the fusiform gyrus, superior temporal gyrus, and inferior parietal cortex. In contrast, reliable intracerebral Accumulation cluster predictors were predominantly located in the superior frontal gyrus, posterior cingulate cortex, and caudal middle frontal gyrus. Fp2 showed similar distributions of intracerebral predictors but with the additional involvement of the supramarginal gyrus in the Accumulation cluster. Predictors of FT9 included middle temporal gyrus regions for the Visual cluster and precentral gyrus for the Accumulation cluster. Finally, predictors for FT10 activity were identified in the PFC pars orbitalis and in inferior temporal regions. The distribution of reliable predictors across clusters (Fig. S5) and the stability of their coefficients across seeds (Fig. S6) are detailed in Supplementary Material, Section 3. Observed differences in proportions between the Visual and Accumulation clusters provide initial insight into the distinct roles these neural processes play within the regression model, shedding light on their respective contributions to the prediction of surface-level ERP signals.

To assess the contribution of each intracerebral cluster to surface ERP signals, we computed the percentage contribution of each cluster by dividing the sum of absolute coefficient values for its reliable predictors by the total sum across all clusters (for details, see Supplementary Material, Section 3). The Accumulation cluster consistently contributed more than the Visual cluster across all four electrodes and all random seeds (see Fig. S7, Supplementary Material). A complementary analysis that included all predictors (both reliable and unreliable) confirmed the same pattern, with the Accumulation cluster consistently contributing more than the Visual cluster. Our interpretation focused on reliable predictors to ensure that conclusions were grounded in stable, replicable patterns in the data.

## Discussion

By combining ERPs, time–frequency analyses, and ridge regression, we aimed to identify how specific patterns of intracerebral neural activity are reflected in scalp EEG signals, thereby bridging the gap between invasive and noninvasive measures of conscious processing.

Our analyses revealed electrophysiological markers of conscious visual target processing, both at the surface EEG level and in corresponding intracerebral signals. Despite the relative paucity of available surface EEG derivations, the analysis of ERPs revealed the presence of reliable markers of conscious perception, particularly within the 350–500 ms time window following target onset. This timing aligns with the latency of the P300 component, a late positive component associated with the conscious perception of relevant sensory stimuli (Polich 2007). This target-related finding was complemented by time–frequency analysis, which revealed differential oscillatory dynamics between consciously and nonconsciously perceived stimuli in the beta and gamma bands preceding target presentation. The increased beta/gamma activity observed at Fp1 for Unseen targets during the pretarget period is reminiscent of magnetoencephalography beta activity observed in the left frontal cortex of patients with right-hemisphere strokes and left neglect who often fail to consciously perceive stimuli on their left side (Rastelli et al. 2013), selectively preceding omissions of responses to left-sided stimuli. This left frontal activity suggests an abnormal prestimulus bias in these patients, reflecting an expectation that no stimulus will appear on the left side (Bartolomeo et al. 2025). In addition, EEG studies in patients with neglect have shown that reductions in the P300 response to contralesional stimuli (Lasaponara et al. 2018, Doricchi et al. 2021) are associated with concomitant alpha desynchronization during the cue period preceding target onset (Lasaponara et al. 2019). These results underscore the role of prestimulus brain states in modulating attention and poststimulus conscious processing. The pretarget beta activity we observed at Fp1 for Unseen targets mirrors this pattern and thus appears to reflect stochastic variations in prestimulus activity specifically associated with the failure to attain a reportable level of stimulus processing. Interestingly, we also found a post-target increase in beta/gamma power at Oz for Seen targets, which may reflect enhanced integration of visual information in occipital areas associated with conscious stimulus processing (Zhu et al. 2022).

When examining intracerebral data, our trajectory-based *k*-means clustering approach identified two predominant neural patterns—Visual and Accumulation clusters—that closely align with the previous study by Liu et al. (2023), which was based on more extensive data. The Visual cluster, characterized by early transient responses (120–340 ms post-target), corresponds to initial sensory processing in visual pathways. The Accumulation cluster, showing gradual activity buildup (230–490 ms post-target), in mainly right-hemisphere frontoparietal networks, likely reflects later integrative mechanisms associated with conscious access and decision-making processes. Our ridge regression analysis revealed that the Accumulation cluster consistently made larger contributions than the Visual cluster in predicting the variance of ERP signals associated with conscious processing. This finding suggests that the later, more sustained neural activity associated with conscious report has a greater impact on the surface-recorded EEG signals than early visual processing. We observed this pattern across all four frontal electrodes (Fp1, Fp2, FT9, FT10), indicating a robust effect that extends beyond specific electrode locations. The anatomical distribution of reliable predictors revealed distinct patterns detailing the diverse cortical networks that contribute to conscious perception, highlighting the role of frontal and higher-order visual processing regions as reliable predictors of conscious report. The finding that Accumulation cluster predictors contribute more strongly to surface EEG signals associated with conscious report than the Visual cluster aligns with theories proposing that consciousness emerges from relatively late, integrative processes rather than early sensory encoding (Bartolomeo et al. 2025). This supports theories such as the GNW, which emphasize the role of sustained, widespread neural activity in conscious access (Dehaene 2001). The limited number of available surface electrodes prevented us from determining whether the signal dynamics more closely corresponded to the P3a or P3b subcomponent of the P300 response. Typically, P3a exhibits a predominantly frontal topography and emerges earlier in the processing stream. It reflects novelty detection and orienting responses to salient stimuli (Polich 2007). In contrast, P3b displays a distinct parietal distribution and appears later in the processing sequence. P3b marks contextual updating processes, the categorization of stimuli based on their match or mismatch to expected ones (Polich 2007, Doricchi et al. 2010, Lasaponara et al. 2018, Doricchi et al. 2021). These components reflect different aspects of conscious processing, with the P3b being associated with conscious access and the global broadcasting of information across brain networks (Dehaene and Changeux 2011, Sergent et al. 2021). While the P3a is likely associated with more automatic attentional capture mechanisms, the P3b is linked to more elaborate cognitive evaluation processes that accompany the conscious processing of stimuli (Polich 2007). This distinction is particularly relevant when investigating the neural correlates of conscious perception, as the P3b component consistently appears in relation to stimuli that reach conscious awareness (Naccache 2018). It should be noted, however, that this interpretation remains debated, with evidence suggesting it may instead reflect residual cognitive processing rather than awareness *per se* (Comanducci et al. 2020).

Given the timing of our observed effects (350–500 ms post-target onset), the ERP component we observed appears to align more closely with the P3b latency than with the P3a. This temporal profile, combined with our focus on the difference between Seen and Unseen target trials—reflecting conscious processing and contextual updating—further supports the involvement of P3b-related processes. We observed significant effects primarily at frontal electrode sites; however, this does not necessarily indicate that we detected a P3a component. Rather, the observed topographical distribution likely reflects the limited spatial sampling of our electrode montage, which did not adequately cover the parietal areas where P3b is typically maximal. It is worth noting that at our occipital (Oz) and temporal derivations (T9, T10), effects were present but did not survive correction for multiple comparisons, possibly due to a lower statistical power at these sites compared to the frontal electrodes. Each of these posterior and temporal sites included fewer participants than the frontal electrodes, potentially obscuring P3b-related activity that may be distributed across both anterior and posterior regions. Our ability to detect the full topographical extent of this activity was constrained by the available electrode coverage. Notwithstanding this methodological limitation, we believe that the results of our study provide important initial insights into the relationship among intracerebral EEG activity, surface EEG activity, and conscious processing in humans.

## Supplementary Material

Lozito_Supplementary_Material_R1_niag011

## Data Availability

The intracranial and surface data that support the findings of this study are available from the corresponding author, S. Lo, upon reasonable request.

## Code availability

The custom codes for trajectory *k*-mean clustering analysis are available at the Github https://github.com/jianghao-liu/attentionconscious-report, with the identifie80 https://doi.org/10.5281/zenodo.8113973 (https://zenodo.org/badge/latestdoi/658009571).
