# Supplementary material for: Towards a bridge between intracerebral and surface EEG signatures of conscious report
Source: Neurosci Conscious. 2026 Apr 8;2026(1):niag011. doi: 10.1093/nc/niag011 (PMC13069880; doi:10.1093/nc/niag011)
Supplement: Lozito_Supplementary_Material_R1_niag011 [file lozito_supplementary_material_r1_niag011.docx]

**Supplementary material for:**

**Towards a Bridge Between Intracerebral and Surface EEG Signatures of Conscious Report**

Silvana Lozito^1,2,3,4*^, Stefano Lasaponara^1,3^, Jianghao Liu^2,5^, Vincent Navarro^6^, Katia Lehongre^7^, Valerio Frazzini^6^, Tal Seidel Malkinson^8#^, Fabrizio Doricchi^1,3#^ & Paolo Bartolomeo^2#^

1 – Department of Psychology – “Sapienza” University of Rome, Italy

2- Sorbonne Université, Institut du Cerveau - Paris Brain Institute - ICM, Inserm, CNRS, AP-HP, Hôpital de la Pitié-Salpêtrière, Paris, France

3 – IRCCS Fondazione Santa Lucia, Rome, Italy

4 – PhD Programme in Behavioural Neuroscience, “Sapienza” University of Rome

5 - Dassault Systèmes, Vélizy-Villacoublay, France.

6 - Epilepsy Unit, AP-HP, PitiéSalpêtrière Hospital, 75013 Paris, France.

7 - CENIR - Centre de Neuro-Imagerie de Recherche, Paris Brain Institute, ICM, Hôpital de la Pitié-Salpêtrière, 75013 Paris, France.

8 - Université de Lorraine, CNRS, IMoPA, F-54000 Nancy, France.

*Corresponding author. e-mail: [silvana.lozito@uniroma1.it](mailto:silvana.lozito@uniroma1.it)

#: Shared senior authorship

**Section S1**

**Linear Mixed Models analyses**

To investigate the neural responses associated with conscious and unconscious visual processing, we employed a Linear Mixed-Effects (LME) model on event-related potentials (ERPs) recorded via surface EEG. This approach was chosen to account for both within-subject variability across trials and between-subject variability. To this aim, we employed the *fitlme* function implemented in MATLAB (MathWorks, 2024a, 2024b), and we were able to preserve the temporal information of each trial without averaging across trials, thereby retaining finer-grained data for statistical analysis.

#### **Model Specification**

The LME model was constructed with ERP amplitude as the response variable. We defined the fixed effects as Condition (seen vs. unseen) and Time (continuous variable representing time points within each trial), as well as their interaction (Condition * Time), to test for differential temporal trends in ERP amplitudes between the two conditions. The inclusion of the interaction term allowed us to assess whether the trajectory of ERP changes over time differs between the seen and unseen conditions, reflecting distinct processing dynamics for conscious and unconscious perception.

The model formula used was:

[
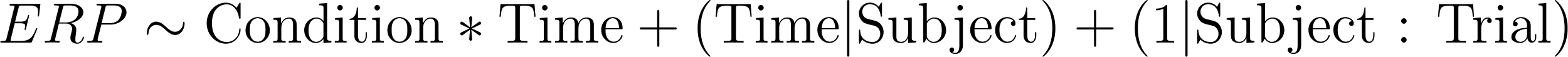
](https://www.codecogs.com/eqnedit.php?latex=%20ERP%20%5Csim%20%5Ctext%7BCondition%7D%20*%20%5Ctext%7BTime%7D%20%2B%20(%5Ctext%7BTime%7D%20%7C%20%5Ctext%7BSubject%7D)%20%2B%20(1%20%7C%20%5Ctext%7BSubject%20%3A%20Trial%7D)%20#0)

where:

1. Conditions and Time are fixed effects.
2. (Time | Subject) represents a random slope of Time within each Subject, accounting for the variability in the temporal response patterns across subjects.
3. (1 | Subject: Trial) is a random intercept for each trial nested within each subject, capturing the trial-level variability within each subject.

#### **Random Effects Structure**

The random effects’ structure allows for inter-individual differences in the ERP temporal profile while controlling for repeated measures within each subject. Specifically:

1. The random intercepts for each trial within each subject [(1 | Subject)] account for any baseline differences in ERP amplitude across trials within the same subject.
2. The random slopes for Time within each Subject [(Time | Subject)] enable the model to accommodate subject-specific temporal trends in ERP responses, acknowledging that each participant might exhibit a unique response profile over time.

These random effects are assumed to follow a normal distribution with a mean of zero. Specifically, for each subject, the random slope associated with Time has a distribution of

[
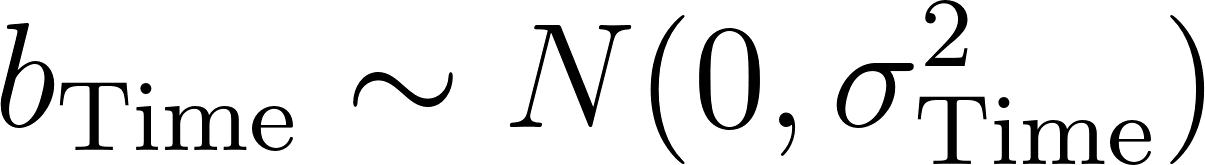
](https://www.codecogs.com/eqnedit.php?latex=%20b_%7B%5Ctext%7BTime%7D%7D%20%5Csim%20N(0%2C%20%5Csigma_%7B%5Ctext%7BTime%7D%7D%5E2)%20#0) capturing the inter-subject variability in the ERP response over time. Additionally, the trial-level random intercepts are modelled as [
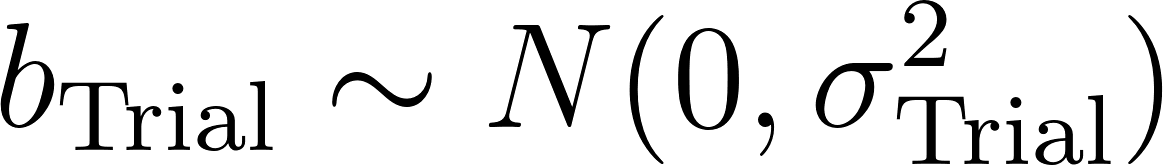
](https://www.codecogs.com/eqnedit.php?latex=%20b_%7B%5Ctext%7BTrial%7D%7D%20%5Csim%20N(0%2C%20%5Csigma_%7B%5Ctext%7BTrial%7D%7D%5E2)%20#0), representing the variability across individual trials within each subject.

####

#### **Model Fitting Procedure**

The model was fitted using MATLAB's fitlme function, with parameters optimized by Maximum Likelihood Estimation (MLE) for both fixed and random effects. The fixed effects coefficients reflect the average ERP amplitude across participants, while the random effects’ structure accounts for individual variability, allowing for a more accurate assessment of Condition and Time effects on ERP amplitude. To ensure flexibility in the model, we explored different covariance structures for the random effects associated with each participant and each trial within a participant. Specifically, we tested configurations that allowed both the temporal slopes (representing changes over time) and intercepts (representing baseline differences) to vary across participants and trials. For each of these elements, we considered both "Full" covariance structures—which allow all elements to be correlated—and "Diagonal" structures, which assume independence between elements while permitting different variances. This approach enabled us to investigate whether simpler, independent variance assumptions or more complex correlations provided a better fit to the data. After testing these configurations, we consistently found no significant interaction effect between Condition and Time, suggesting that condition-related differences in ERP amplitude did not emerge or evolve significantly over time. This analysis was conducted across multiple time windows, including the cue period, the post-target period, and specific windows associated with the P300 component ([350, 500],[400,500]).

Given the lack of significant findings from the linear mixed model analysis, we proceeded to inspect the ERP waveforms at the individual participant level. This step revealed that some participants, particularly in the post-target period, exhibited slightly different ERP components compared to others. Even though a visual inspection of the averaged ERP plot across participants suggested the presence of an effect, especially within the 350–500 ms post-target window, this was not supported by statistical analysis. This discrepancy could be attributed to the limited statistical power of the study, as the small sample size and high inter-subject variability likely obscured the ability to detect a significant effect.

**Section S2**

**Trajectory k-means clustering**


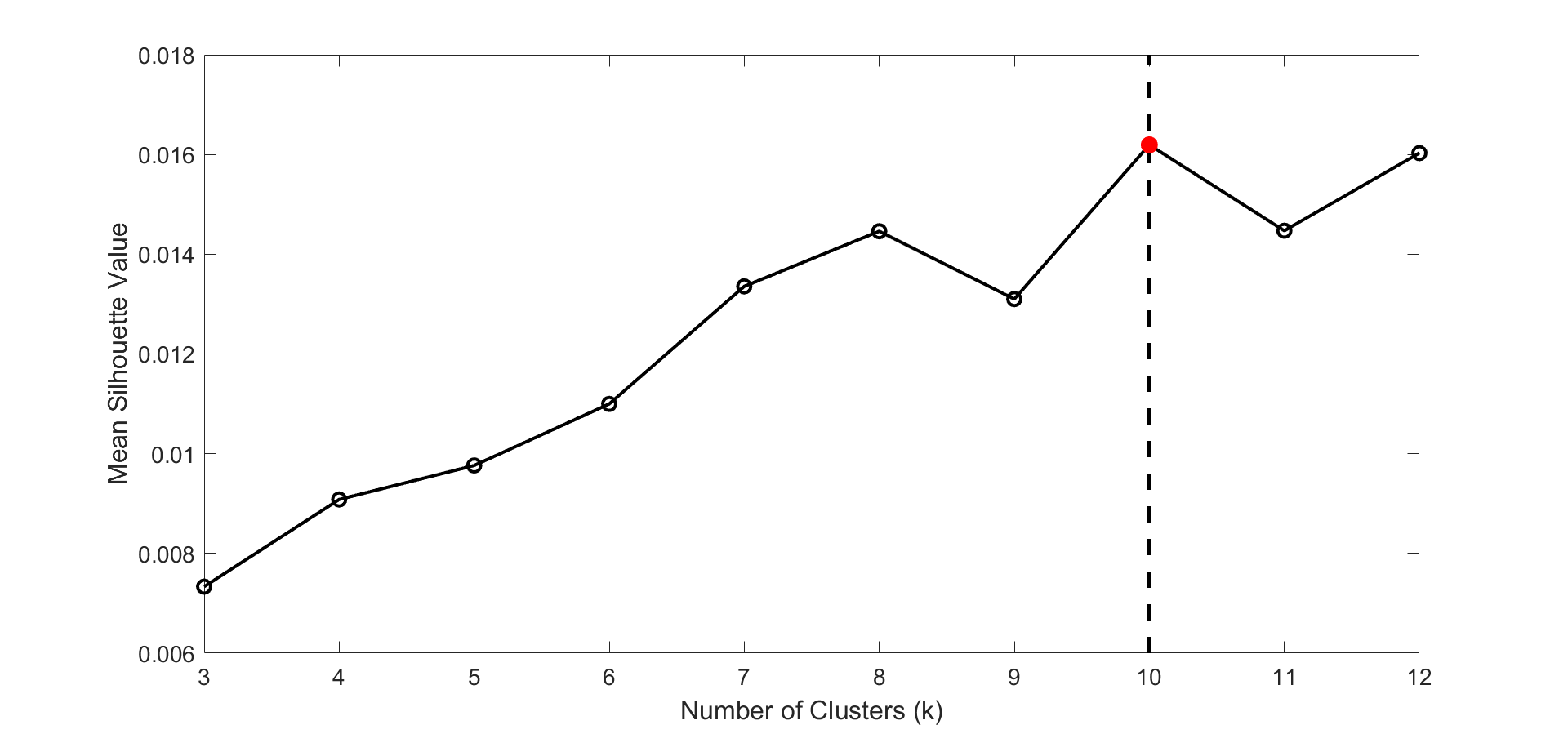


**Figure S1**: Mean silhouette values for k-means clustering solutions across different numbers of clusters (k). The red dot indicates the highest silhouette value, corresponding to k=10, suggesting the best balance between cluster cohesion and separation.

###
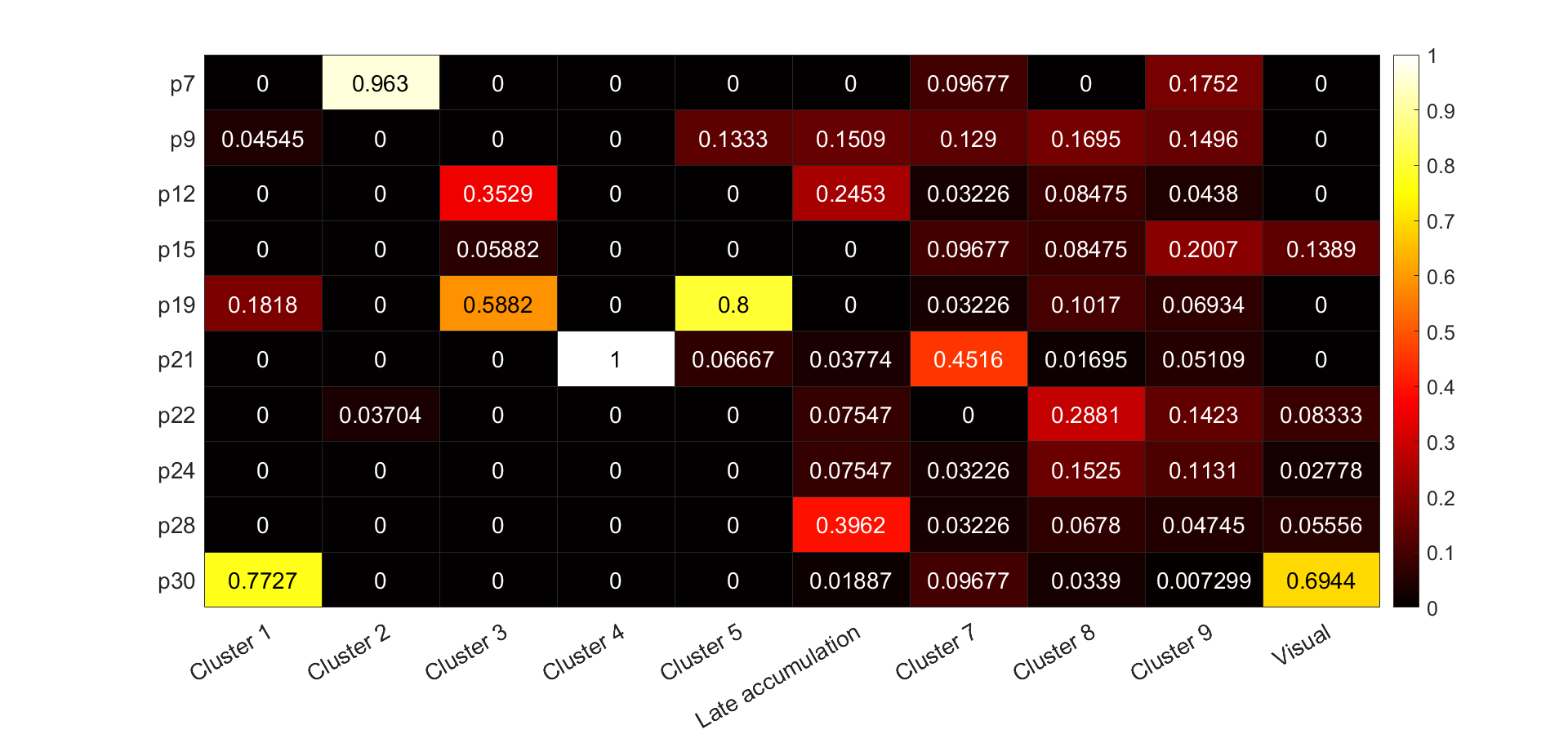


**Figure S2**: Proportion of cluster contacts per patient. Cluster 6 is the Late accumulation cluster, and Cluster 10 is the Visual cluster.


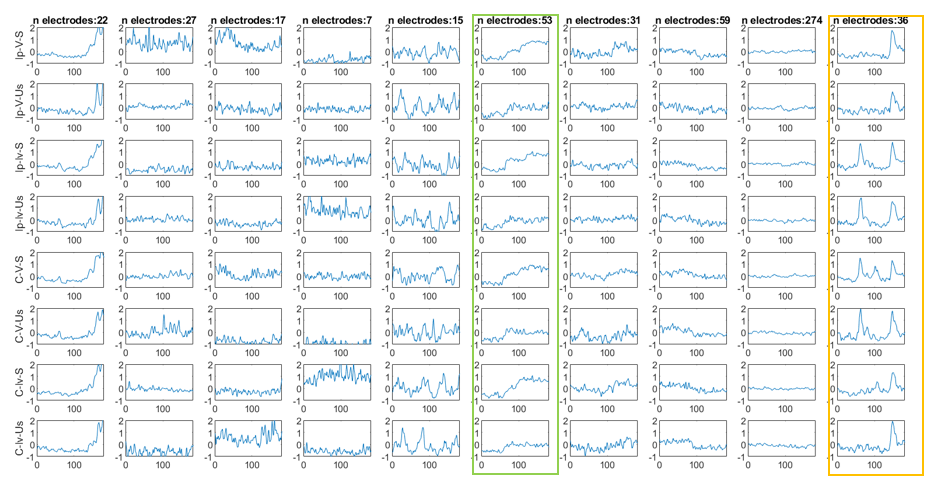


**Figure S3:** Temporal dynamics of HFBB activity for each cluster identified in the k-means clustering analysis with k = 10. The cluster highlighted in green represents the *late accumulation pattern*, while the cluster highlighted in orange corresponds to the *visual pattern*. The number of electrodes contributing to each cluster is shown at the top of each column.

**Section S3**

**Ridge regression**

**Optimization problem**

Ridge regression is a method for estimating coefficients of linear models that include linearly correlated predictors (MathWorks, 2024c). Coefficient estimates for multiple linear regression models rely on the independence of the model terms. When terms are correlated, and the columns of the design matrix X have an approximately linear dependence, the matrix [
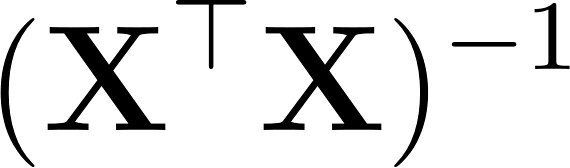
](https://www.codecogs.com/eqnedit.php?latex=%20(%5Cmathbf%7BX%7D%5E%5Ctop%20%5Cmathbf%7BX%7D)%5E%7B-1%7D%20#0) is close to singular. Therefore, the least-squares estimate

[
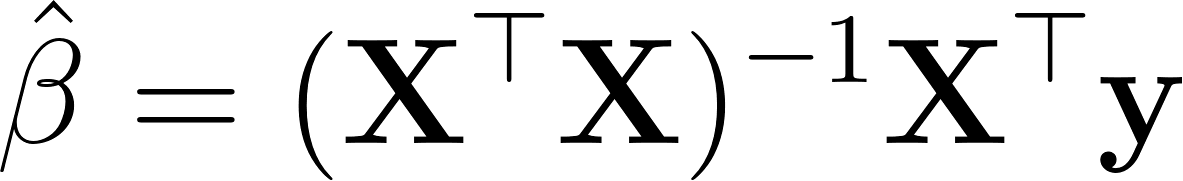
](https://www.codecogs.com/eqnedit.php?latex=%5Chat%7B%5Cbeta%7D%20%3D%20(%5Cmathbf%7BX%7D%5E%5Ctop%20%5Cmathbf%7BX%7D)%5E%7B-1%7D%20%5Cmathbf%7BX%7D%5E%5Ctop%20%5Cmathbf%7By%7D#0)

is highly sensitive to random errors in the observed response *y*, producing a large variance.

Ridge regression addresses the problem of multicollinearity by estimating regression coefficients using

[
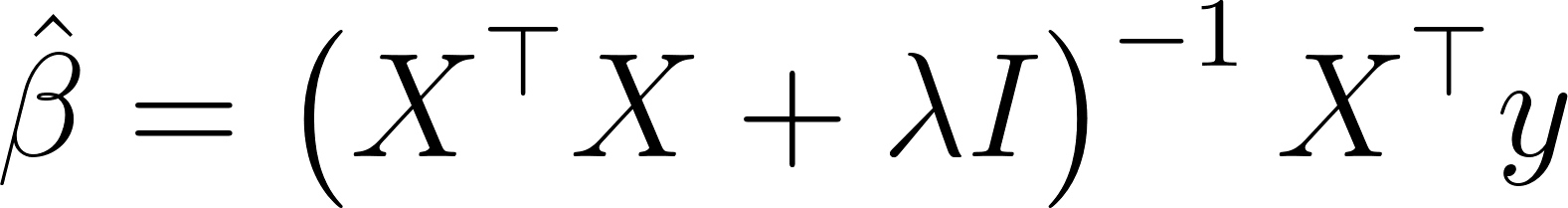
](https://www.codecogs.com/eqnedit.php?latex=%5C%20%5Chat%7B%5Cbeta%7D%20%3D%20%5Cleft(%20X%5E%5Ctop%20X%20%2B%20%5Clambda%20I%20%5Cright)%5E%7B-1%7D%20X%5E%5Ctop%20y%20%5C#0)

where [
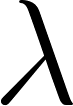
](https://www.codecogs.com/eqnedit.php?latex=%20%5Clambda%20#0) is the ridge parameter and *I* is the identity matrix. Small, positive values of [
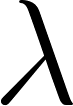
](https://www.codecogs.com/eqnedit.php?latex=%20%5Clambda%20#0) improve the conditioning of the problem and reduce the variance of the estimates. While biased, the reduced variance of ridge estimates often results in a smaller mean squared error when compared to least-squares estimates. For a given value of a nonnegative parameter, Ridge solves the following optimization problem:

[
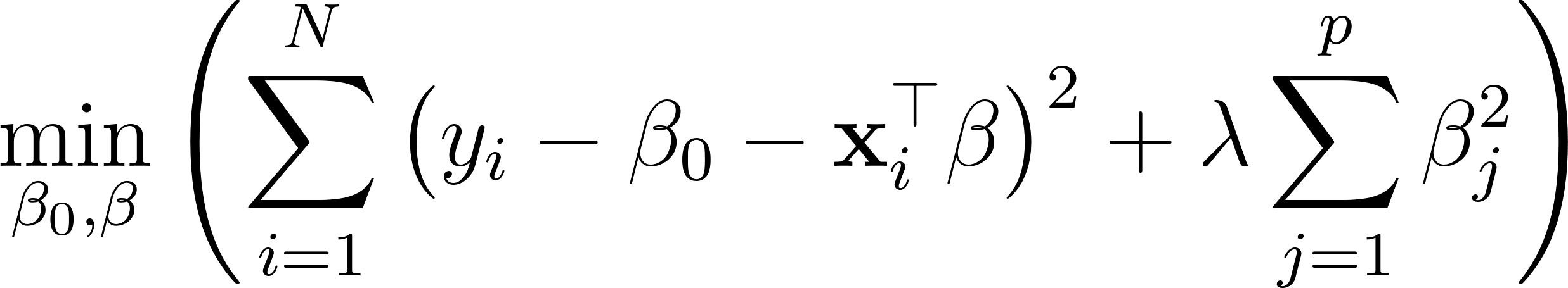
](https://www.codecogs.com/eqnedit.php?latex=%5Cmin_%7B%5Cbeta_0%2C%20%5Cbeta%7D%20%5Cleft(%20%5Csum_%7Bi%3D1%7D%5EN%20%5Cleft(%20y_i%20-%20%5Cbeta_0%20-%20%5Cmathbf%7Bx%7D_i%5E%5Ctop%20%5Cbeta%20%5Cright)%5E2%20%2B%20%5Clambda%20%5Csum_%7Bj%3D1%7D%5Ep%20%5Cbeta_j%5E2%20%5Cright)#0)

where:

- [
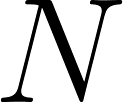
](https://www.codecogs.com/eqnedit.php?latex=%20N%20#0) is the number of observations,
- [
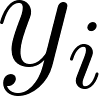
](https://www.codecogs.com/eqnedit.php?latex=%20y_i%20#0) is the response at observation [
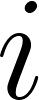
](https://www.codecogs.com/eqnedit.php?latex=%20i%20#0),
- [
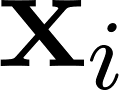
](https://www.codecogs.com/eqnedit.php?latex=%20%5Cmathbf%7Bx%7D_i%20#0) is the data, a vector of length [
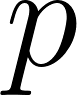
](https://www.codecogs.com/eqnedit.php?latex=%20p%20#0) at observation [
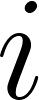
](https://www.codecogs.com/eqnedit.php?latex=%20i%20#0),
- [
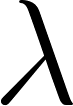
](https://www.codecogs.com/eqnedit.php?latex=%20%5Clambda%20#0) is a nonnegative regularization parameter corresponding to one value of [
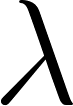
](https://www.codecogs.com/eqnedit.php?latex=%20%5Clambda%20#0),
- [
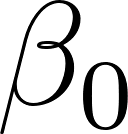
](https://www.codecogs.com/eqnedit.php?latex=%20%5Cbeta_0%20#0) is a scalar, and [
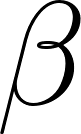
](https://www.codecogs.com/eqnedit.php?latex=%20%5Cbeta%20#0) is a vector of length [
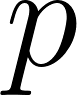
](https://www.codecogs.com/eqnedit.php?latex=%20p%20#0).

**K-fold cross-validation**

To determine the optimal regularization parameter ([**
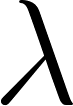
**](https://www.codecogs.com/eqnedit.php?latex=%5Clambda#0)) for each electrode, we employed 10-fold cross-validation, a robust method for balancing model complexity and prediction accuracy. This technique involves splitting the dataset into 10 subsets (folds), where nine folds are used for training and the remaining one for testing. This process is iterated until every fold has been used as a testing set once, ensuring that the evaluation is both exhaustive and unbiased. The mean squared error (MSE) was computed for a wide range (100) of [**
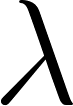
**](https://www.codecogs.com/eqnedit.php?latex=%5Clambda#0) values, spanning logarithmically from [
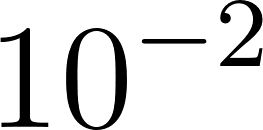
](https://www.codecogs.com/eqnedit.php?latex=10%5E%7B-2%7D#0) to [
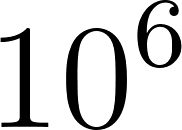
](https://www.codecogs.com/eqnedit.php?latex=10%5E6#0). This broad search allowed us to explore the impact of different regularization strengths. With low regularization, coefficients tend to exhibit greater variability and larger magnitudes, potentially reflecting overfitting. Conversely, high regularization risks underfitting due to excessive smoothing of coefficients. These dynamics highlight how the regularization parameter influences the behaviour of ridge regression coefficients, shaping their variability and magnitude. The value of [**
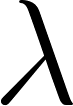
**](https://www.codecogs.com/eqnedit.php?latex=%5Clambda#0) minimizing the mean MSE across the folds was selected as the optimal one for each electrode. To ensure robustness, this procedure was repeated using 10 random seeds. These seeds were generated randomly but then saved and consistently applied across the analysis of all surface electrodes. This approach guaranteed reproducibility while allowing us to evaluate the stability of the results across different random partitions of the dataset, ensuring that the identified patterns were not dependent on a single initialization. For each seed, the MSE curve was computed across all 100 values of [**
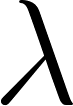
**](https://www.codecogs.com/eqnedit.php?latex=%5Clambda#0), and the corresponding optimal [**
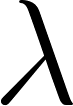
**](https://www.codecogs.com/eqnedit.php?latex=%5Clambda#0) was identified. By aggregating the MSE curves corresponding to these optimal [**
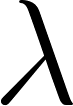
**](https://www.codecogs.com/eqnedit.php?latex=%5Clambda#0) values, we calculated the mean MSE curve (solid line) and its variability across seeds (shaded areas representing ±1 standard deviation). This approach provides insights into the average performance of the model for the set of optimal [**
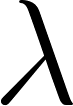
**](https://www.codecogs.com/eqnedit.php?latex=%5Clambda#0) values, as well as the variability induced by the random partitioning of the data.

**Bootstrapped confidence intervals**

To assess the robustness and reliability of predictors in their contributions to the regression model, confidence intervals (CIs) for the regression coefficients were computed using a bootstrap approach (Özkale & Altuner, 2023). This methodology accounts for variability introduced by resampling, providing a measure of the stability and robustness of predictor contributions under ridge regression regularization. For each of the 10 random seeds, 1000 bootstrap samples were generated by resampling observations (data points) from the original dataset with replacement. For each bootstrap sample, ridge regression was applied using the seed-specific optimal regularization parameter (​[**
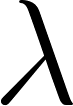
**](https://www.codecogs.com/eqnedit.php?latex=%5Clambda#0)), and the corresponding regression coefficients were estimated. This process produced a distribution of bootstrap-derived coefficients for each predictor, reflecting the empirical variability of the estimates under resampling. At a 95% confidence level, the CIs were determined by extracting the 2.5th and 97.5th percentiles of the bootstrapped coefficient distributions. This percentile-based approach ensured that the CIs captured the true variability of each predictor’s coefficients while remaining consistent with the regularization effects inherent to the ridge regression framework. Importantly, predictors whose confidence intervals did not include zero were deemed reliable if they were consistently identified as such across all seeds, as their contributions were unlikely to result from random fluctuations. This criterion allowed for the identification of predictors with consistent effects across bootstrap samples.

**Quantification of Cluster Contributions Across Seeds**

We analysed the contribution of the identified intracranial clusters to the prediction of surface-level ERP signals by summing the absolute values of the coefficients of reliable predictors within each cluster ([
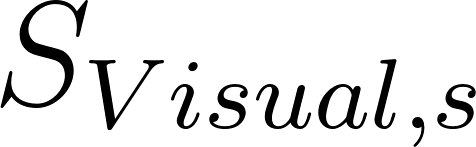
](https://www.codecogs.com/eqnedit.php?latex=S_%7BVisual%2C%20s%7D#0)​,[
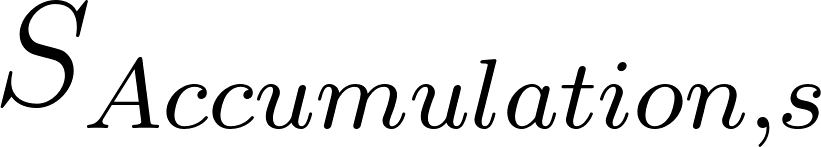
](https://www.codecogs.com/eqnedit.php?latex=S_%7BAccumulation%2C%20s%7D#0)​) and dividing these sums by the total sum of the absolute values of the coefficients of all reliable predictors across both clusters ([
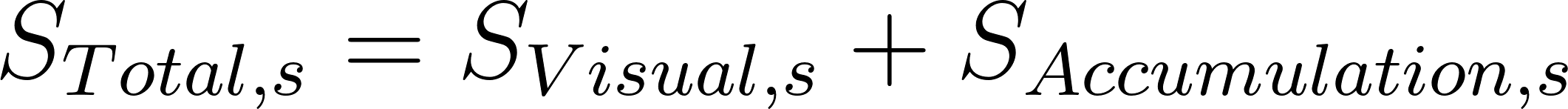
](https://www.codecogs.com/eqnedit.php?latex=S_%7BTotal%2C%20s%7D%20%3D%20S_%7BVisual%2C%20s%7D%20%2B%20S_%7BAccumulation%2C%20s%7D#0)) and expressed as percentages:

[
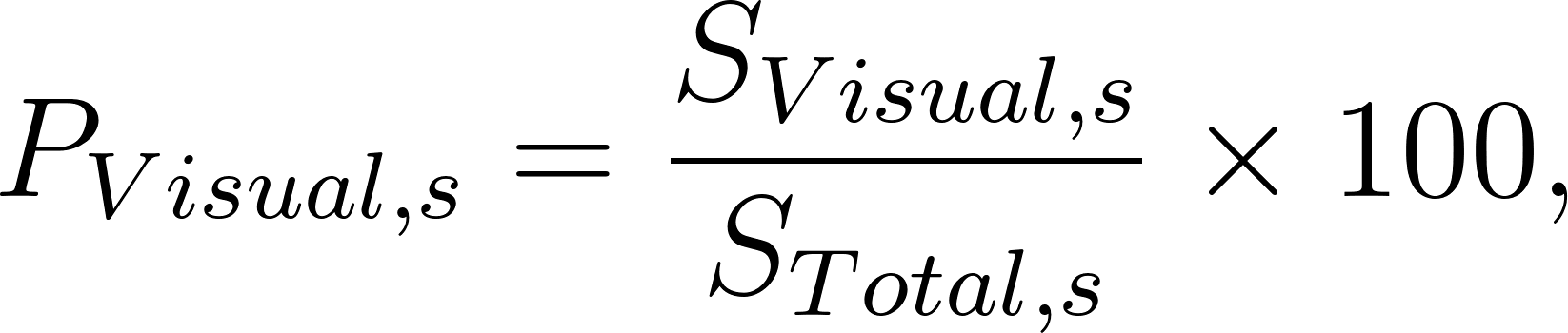
](https://www.codecogs.com/eqnedit.php?latex=P_%7BVisual%2C%20s%7D%20%3D%20%5Cfrac%7BS_%7BVisual%2C%20s%7D%7D%7BS_%7BTotal%2C%20s%7D%7D%20%5Ctimes%20100%2C%20%5Cquad%20#0) [
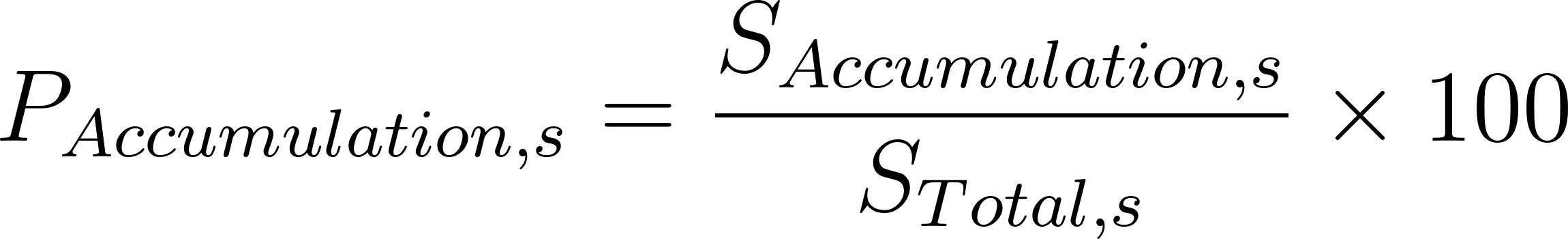
](https://www.codecogs.com/eqnedit.php?latex=P_%7BAccumulation%2C%20s%7D%20%3D%20%5Cfrac%7BS_%7BAccumulation%2C%20s%7D%7D%7BS_%7BTotal%2C%20s%7D%7D%20%5Ctimes%20100%20#0)

This approach was applied for each of the 10 seeds, providing insight into how much each cluster contributes to the overall prediction when considering only reliable predictors. Here, [
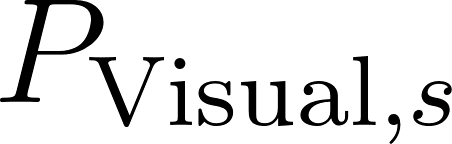
](https://www.codecogs.com/eqnedit.php?latex=P_%7B%5Ctext%7BVisual%7D%2Cs%7D#0) and [
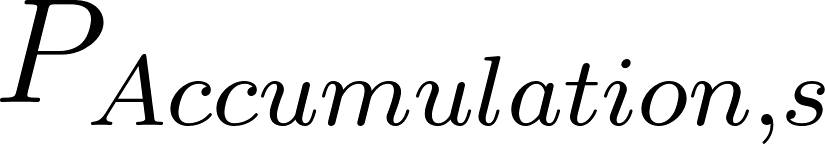
](https://www.codecogs.com/eqnedit.php?latex=P_%7BAccumulation%2C%20s%7D#0) represent the percentage contributions of the Visual and Accumulation clusters, respectively, for each seed [
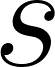
](https://www.codecogs.com/eqnedit.php?latex=s#0). This approach allowed us to account for variability across seeds while focusing on the subset of predictors deemed reliable (i.e., those whose bootstrap confidence intervals excluded zero across all seeds). Additionally, we assessed overall reliability by comparing the contribution of reliable predictors to the total contribution of all predictors, both reliable and unreliable. This comparison highlights the robustness of the reliable predictors in the context of all potential predictors. This additional analysis contextualized the contribution of reliable predictors against the full set of predictors, providing a more comprehensive view of how reliably Visual and Accumulation predictors contributed to the model's overall explanatory power. By examining the consistency of cluster contributions across both methods and seeds, we aimed to determine whether the different contributions of the clusters remained stable, ensuring that our findings were not dependent on a specific analytical approach or seed.


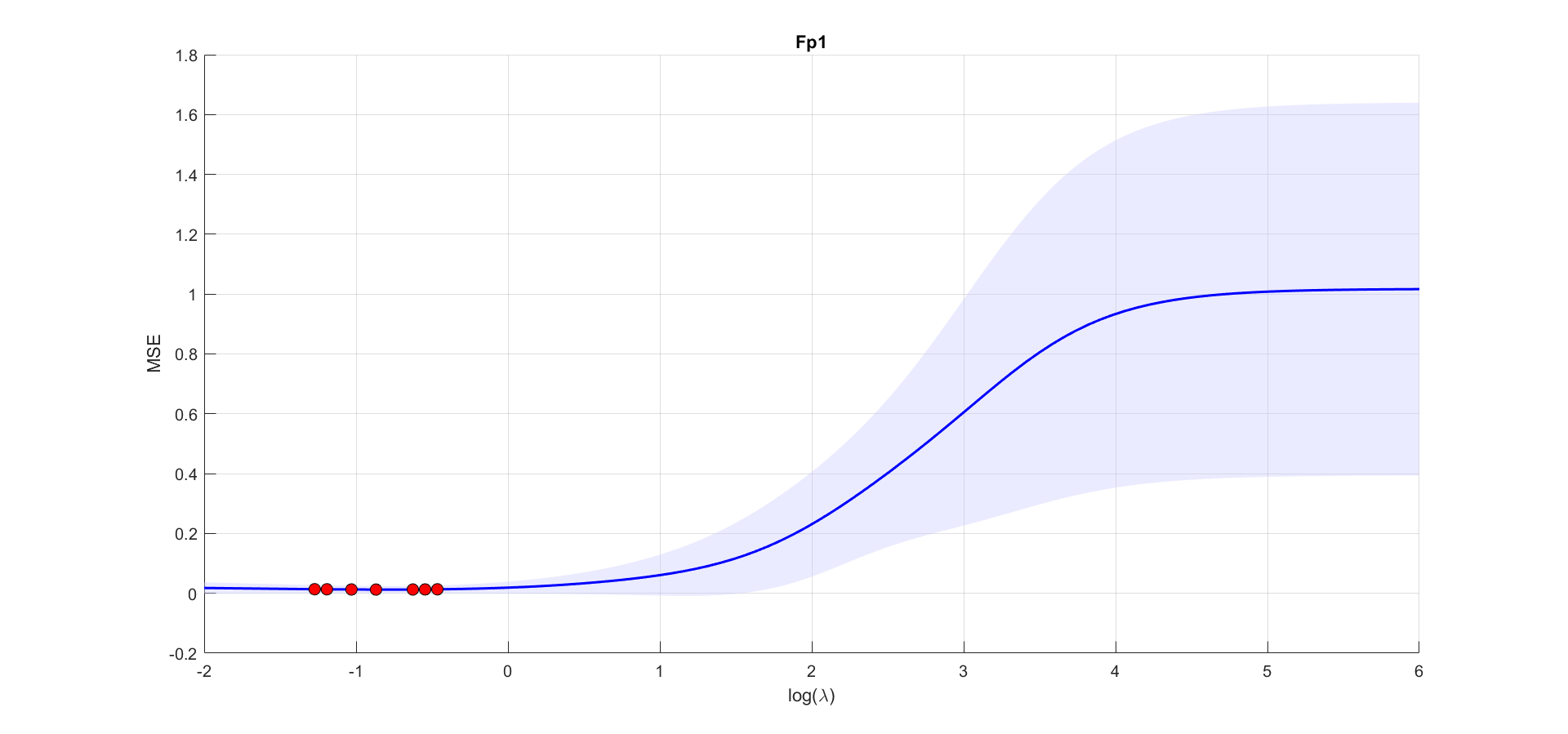

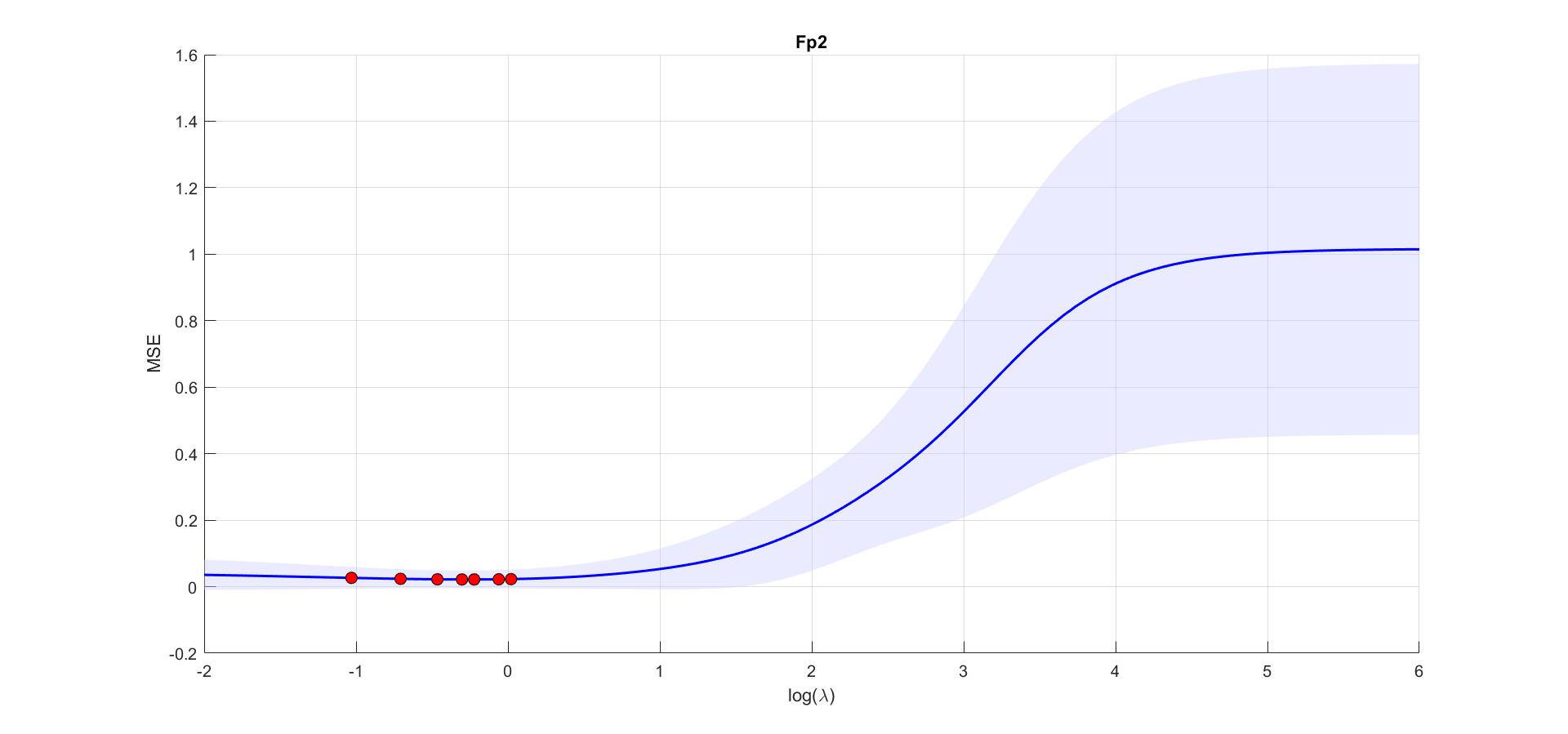

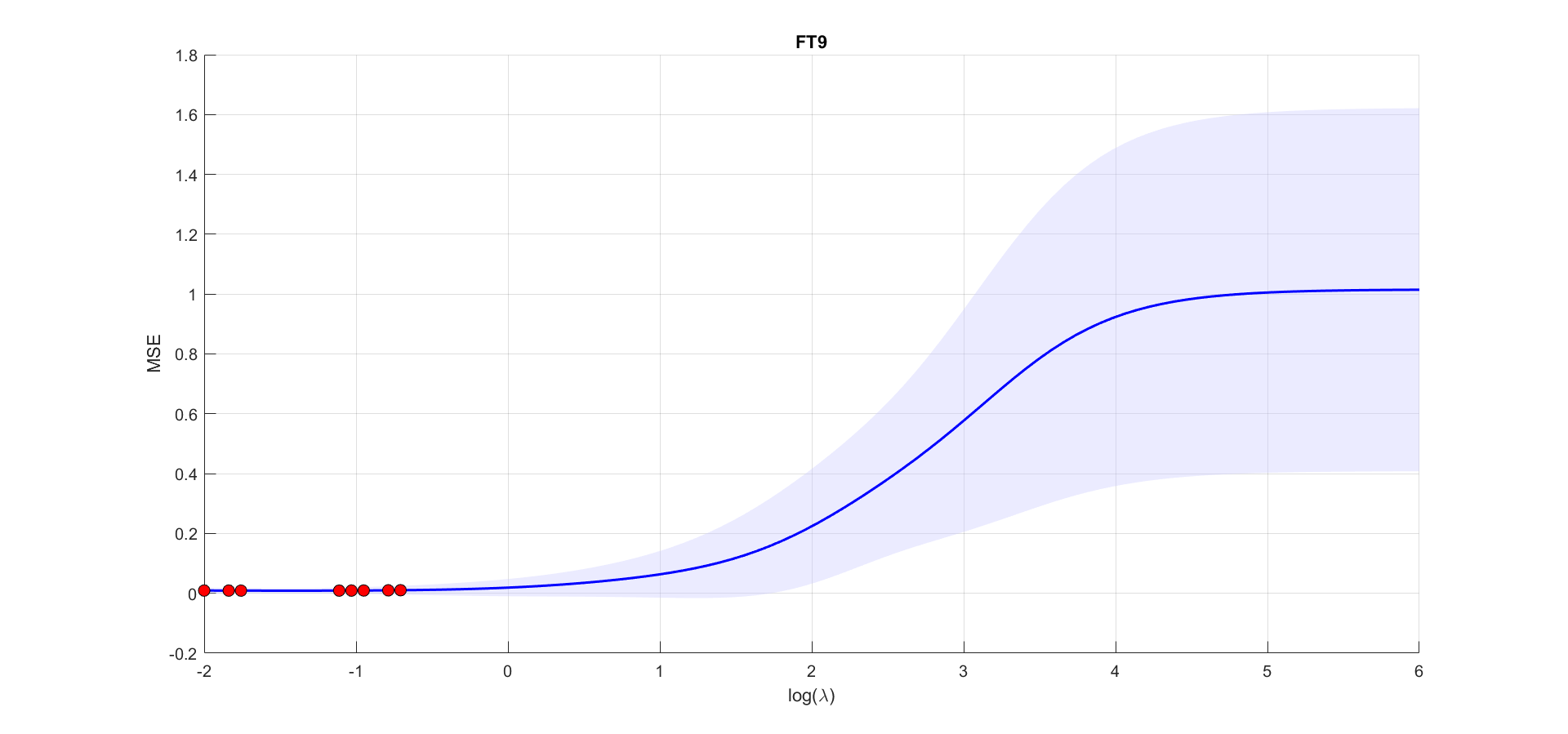

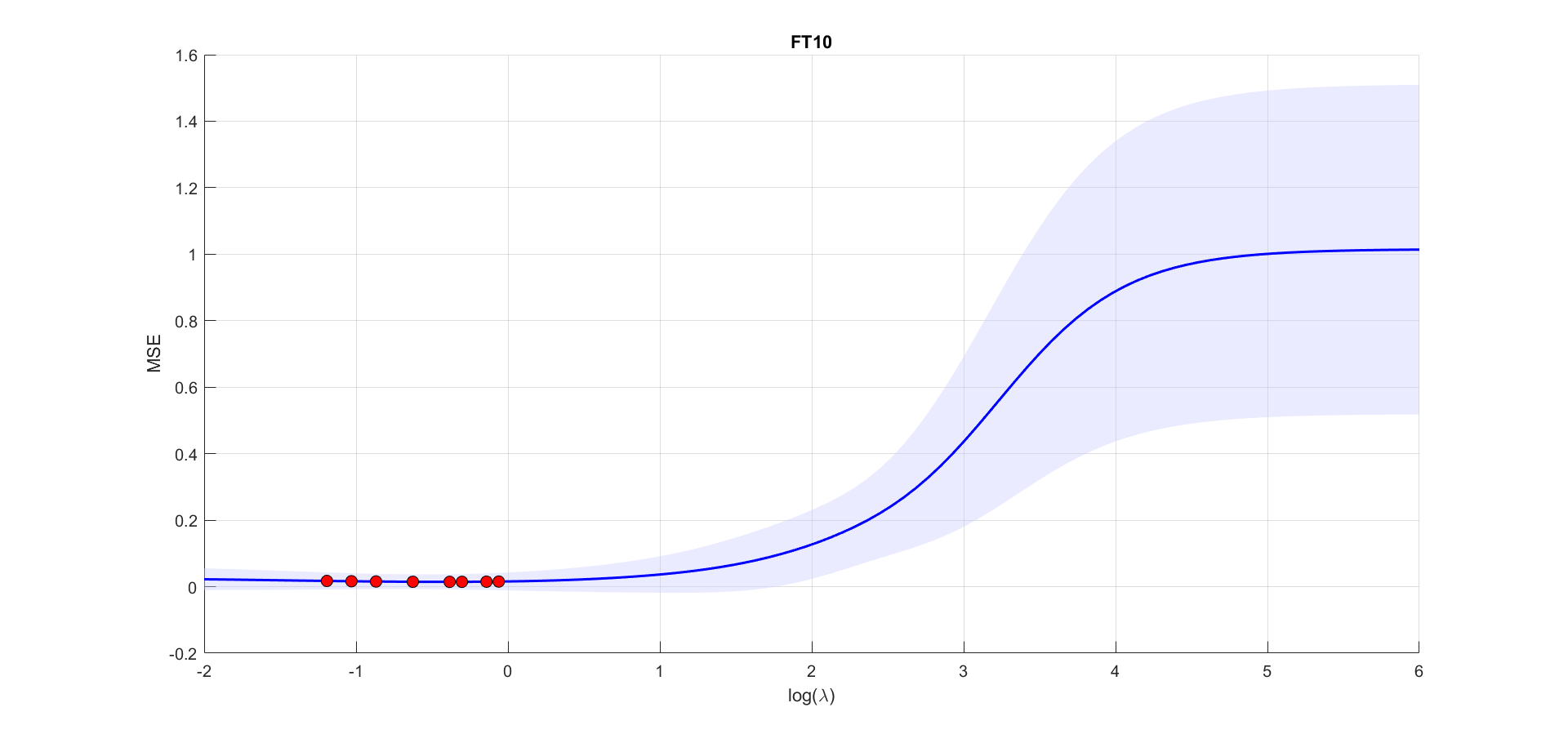


**Figure S4:** Mean MSE trends as a function of log(λ) for electrodes Fp1, Fp2, FT9, and FT10. For each seed, the MSE is averaged across 10 folds of cross-validation. Blue lines represent the average of these 10 MSE curves (one per seed), while the shaded areas show the variability across the 10 seeds (±1 standard deviation from the mean). Red dots indicate the λopt​ values obtained for each random seed in the cross-validation process. If fewer than 10 markers are visible, it is because some values coincide.


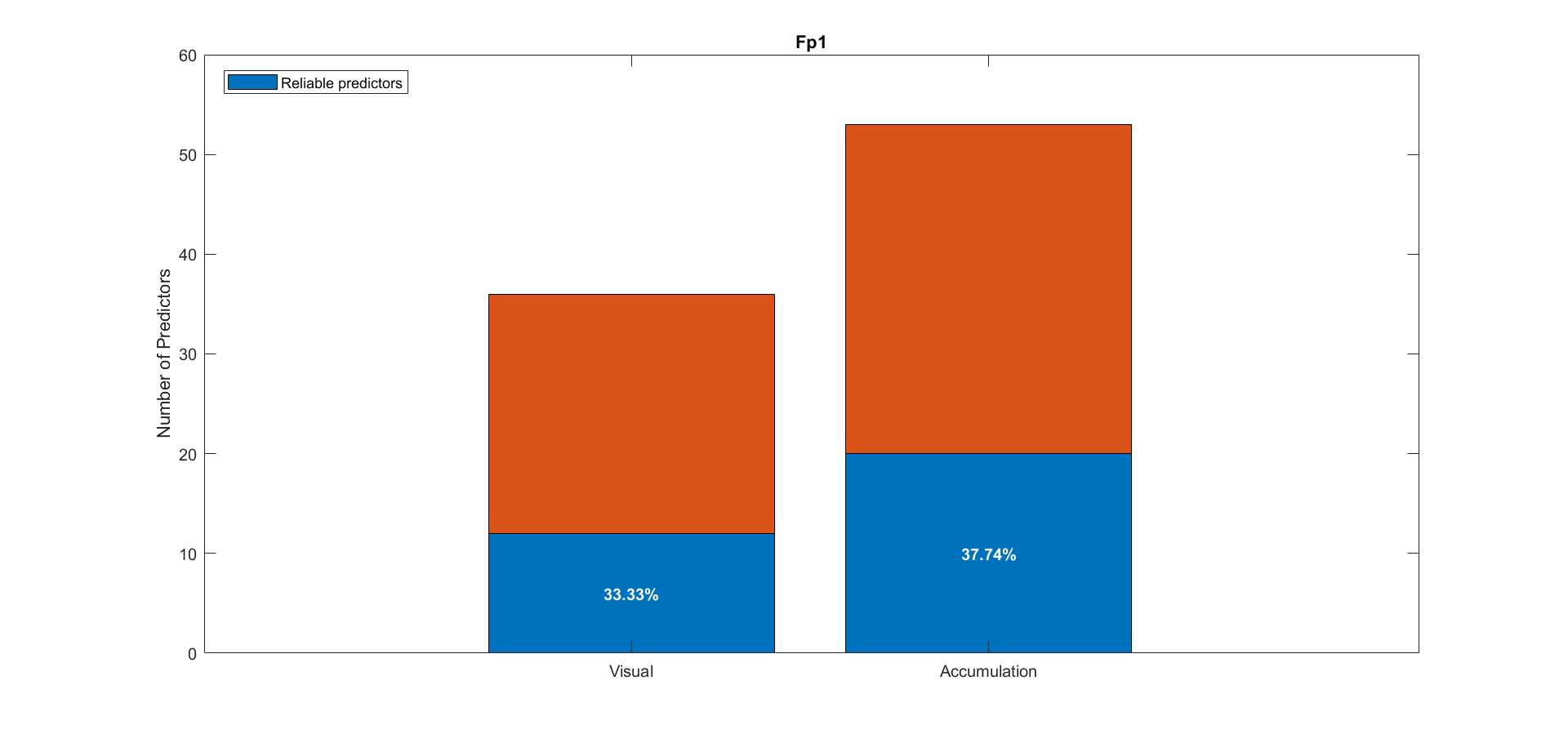

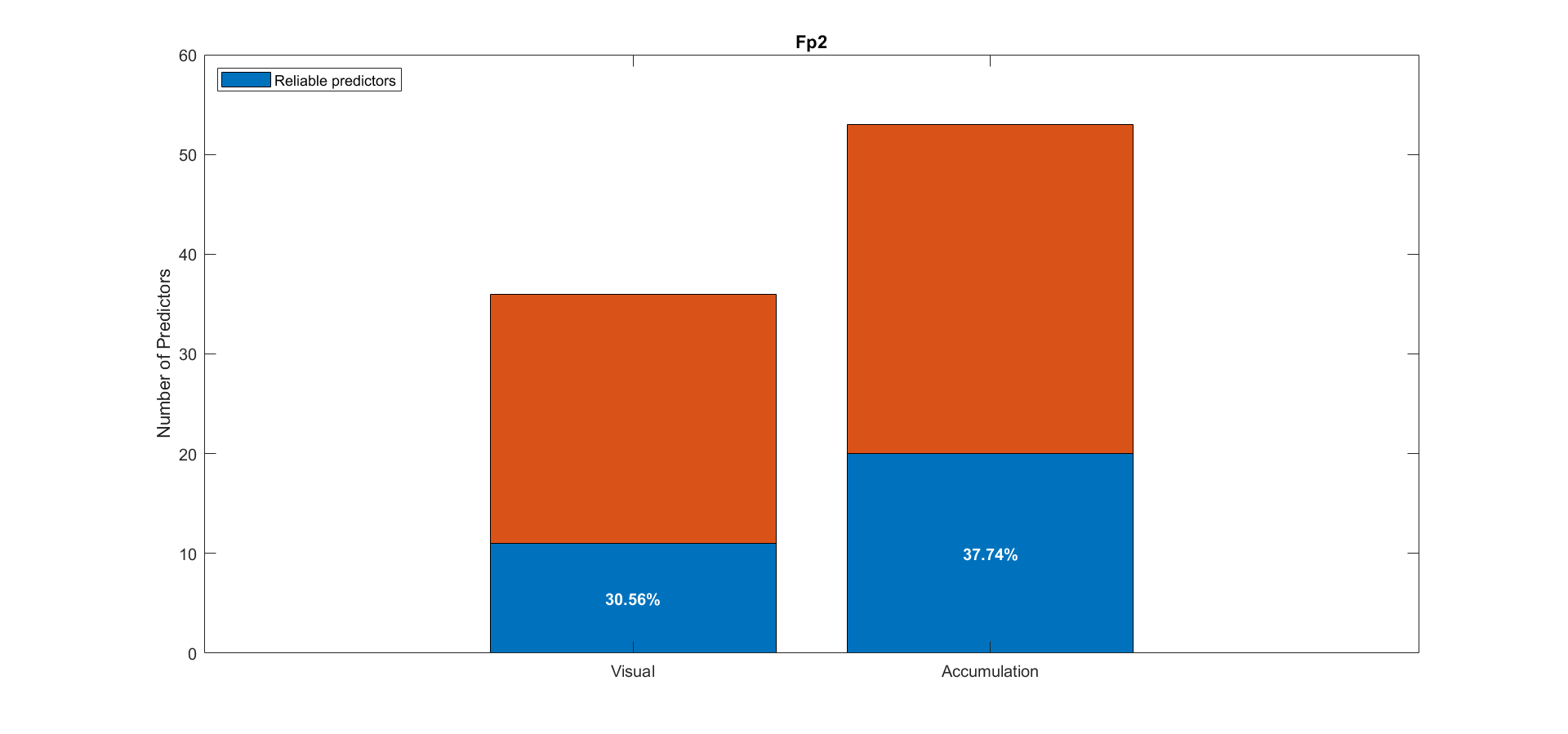


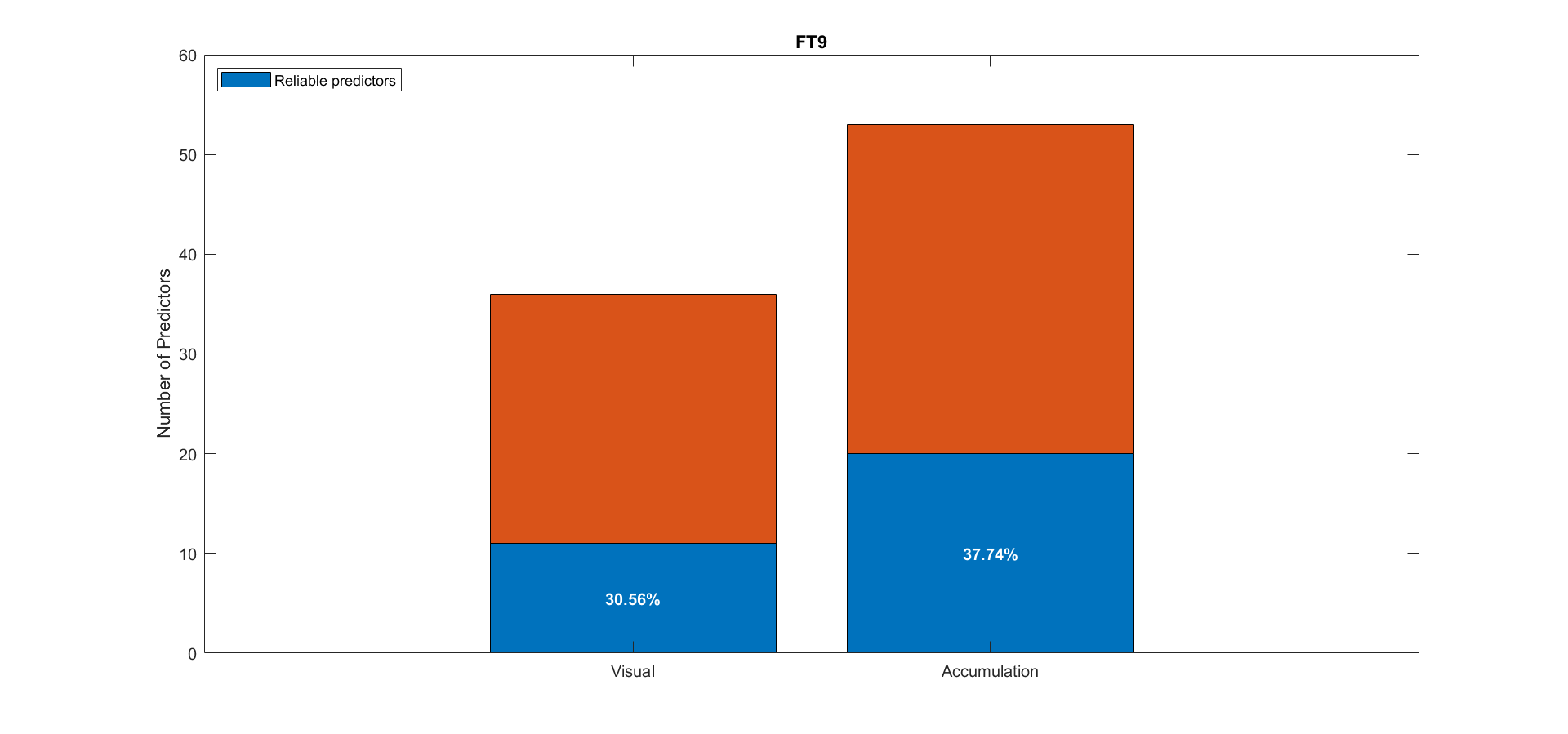

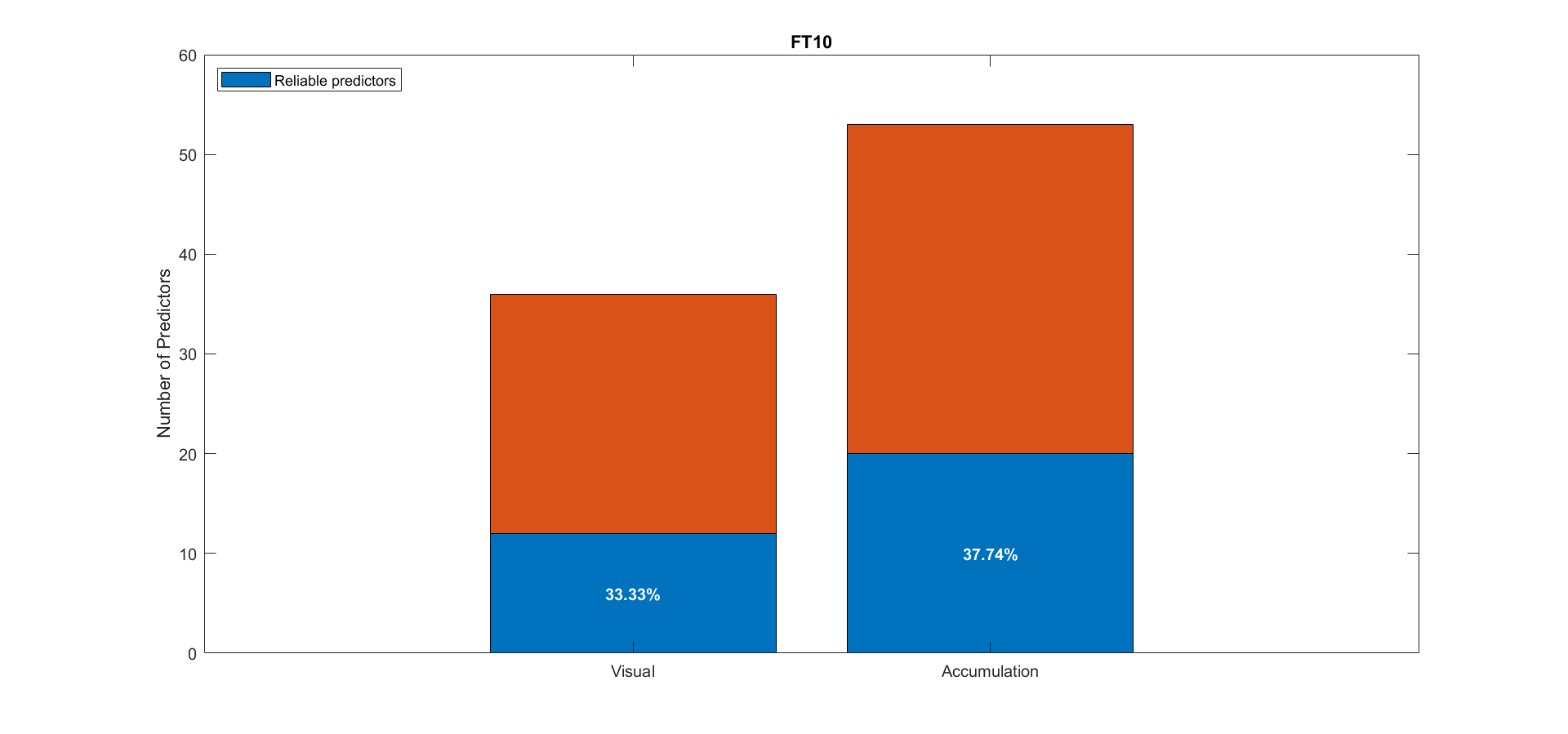


**Figure S5:**  Bar plots representing, in their total height (including the orange portion), the total number of predictors in each of the two clusters (Visual and Accumulation) for the four surface electrodes (Fp1, Fp2, FT9, FT10). The blue portion of each bar indicates the number of reliable predictors (across all 10 seeds), with the percentage shown representing the proportion of reliable predictors relative to the total number of predictors within each cluster. Observed differences in the proportions between the Visual and Accumulation clusters provide a first insight into the distinct roles these neural processes play within the regression model, shedding light on their respective contributions to the prediction of surface-level ERP signals.


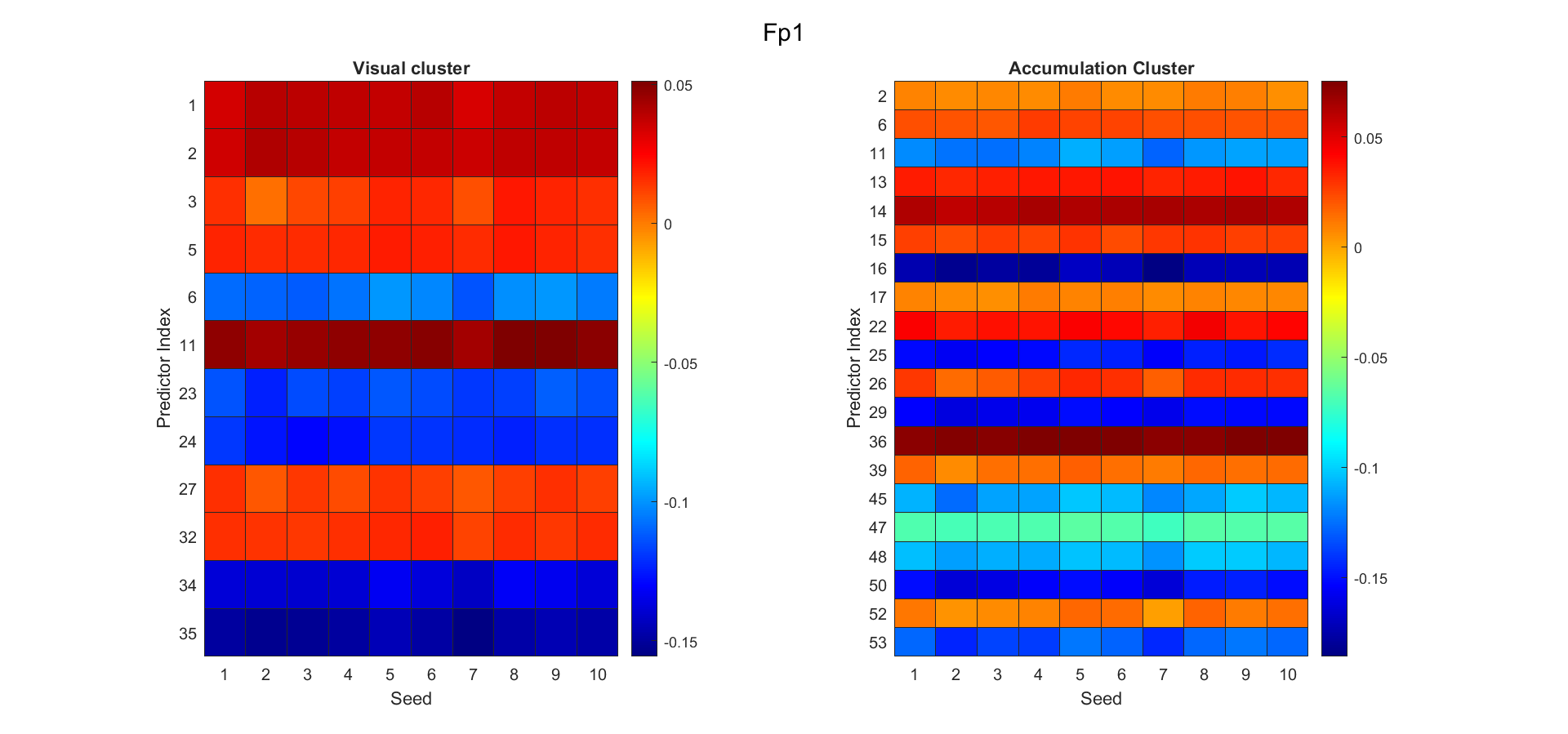


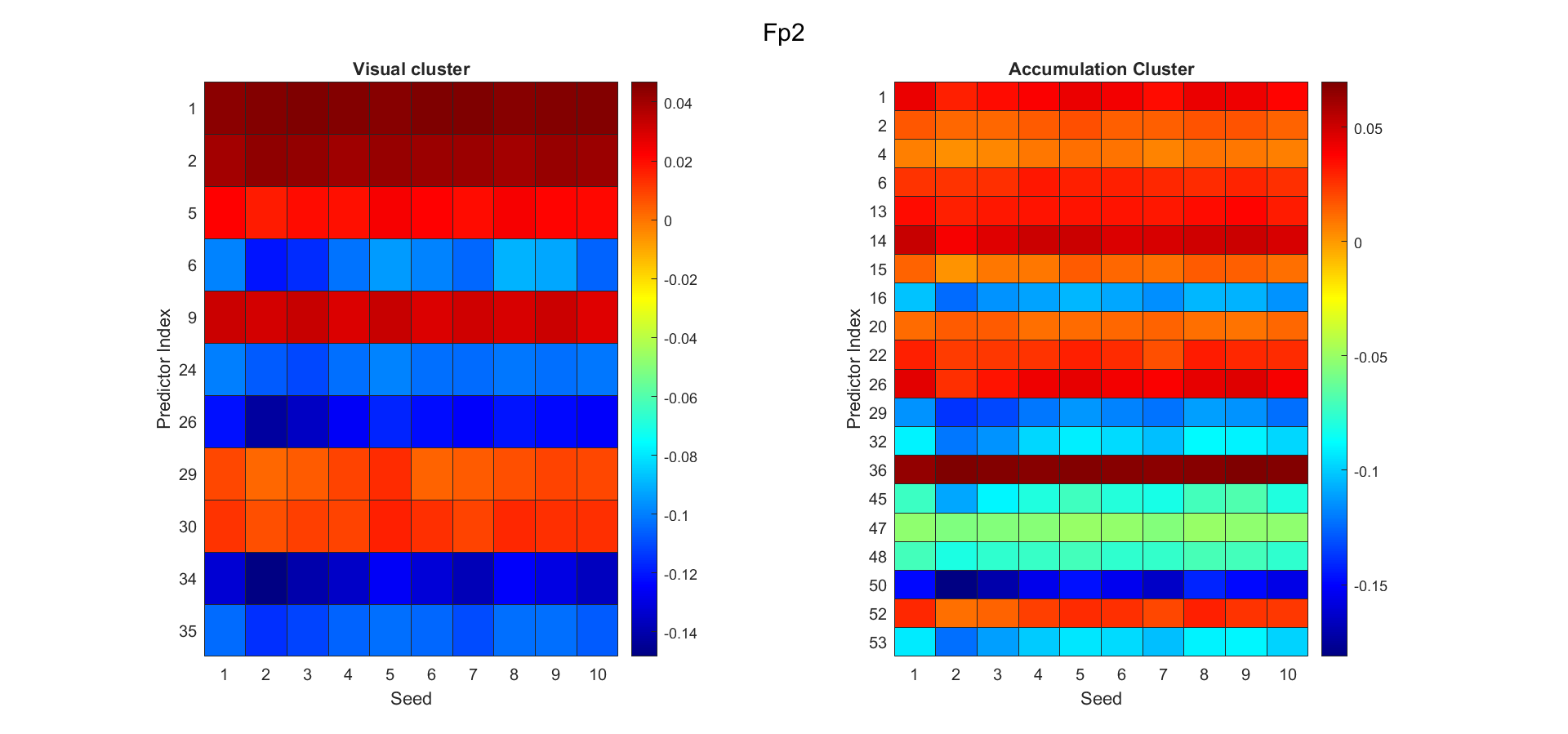


z


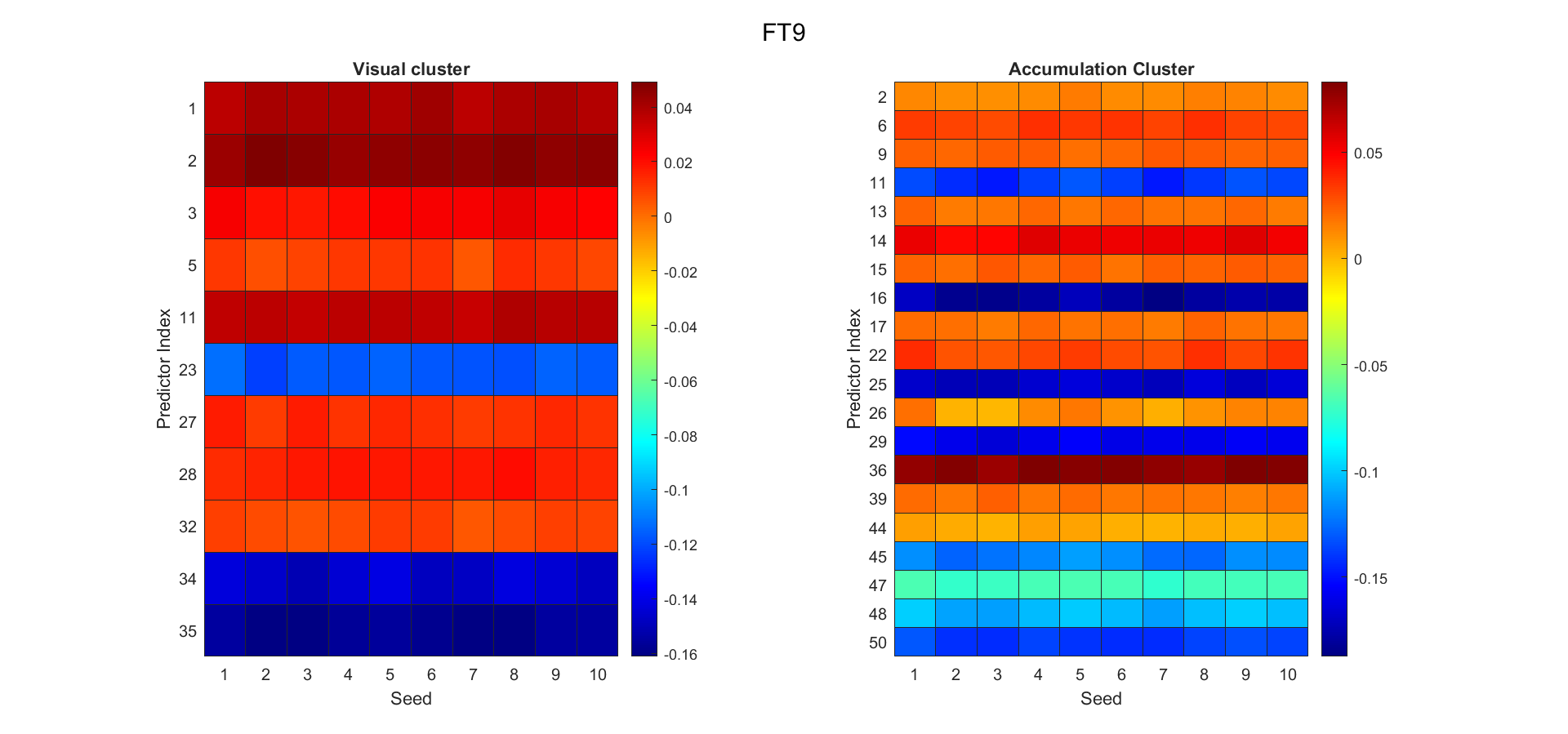


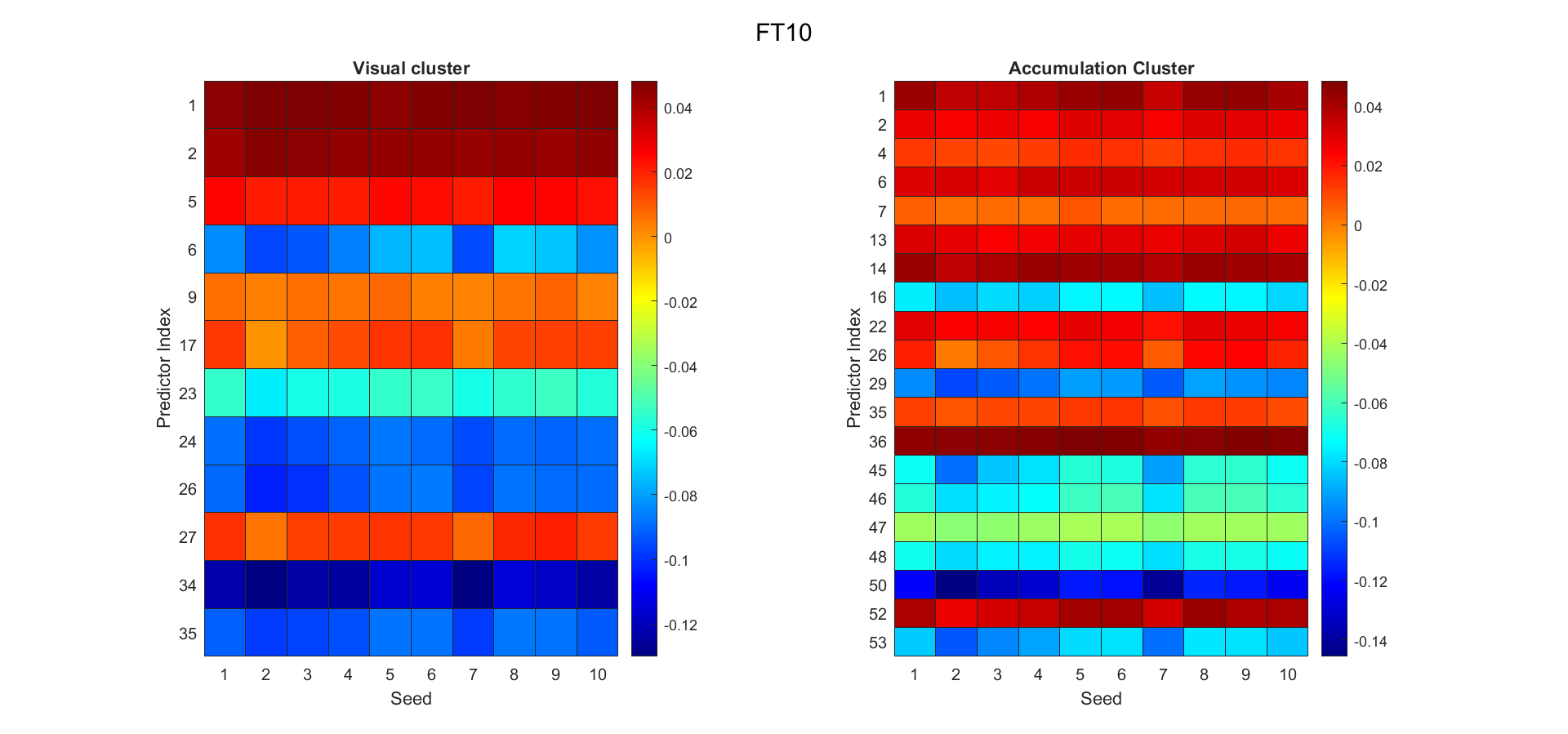


**Figure S6:** Heatmaps showing the coefficients of reliable predictors for the Visual (left panel) and Accumulation (right panel) clusters for Fp1, Fp2, FT9, and FT10. Each row corresponds to a specific predictor index within the respective cluster, while each column represents one of the 10 seed iterations used for the analysis. The color scale reflects the magnitude of the coefficients, with red indicating higher positive values and blue indicating higher negative values. These heatmaps provide a detailed visualization of variability and stability in predictor contributions across seeds. Predictors with consistent coefficients across seeds appear as uniformly coloured rows, indicating robust reliability. In contrast, predictors with high variability in coefficients exhibit heterogeneity in color across their corresponding rows.


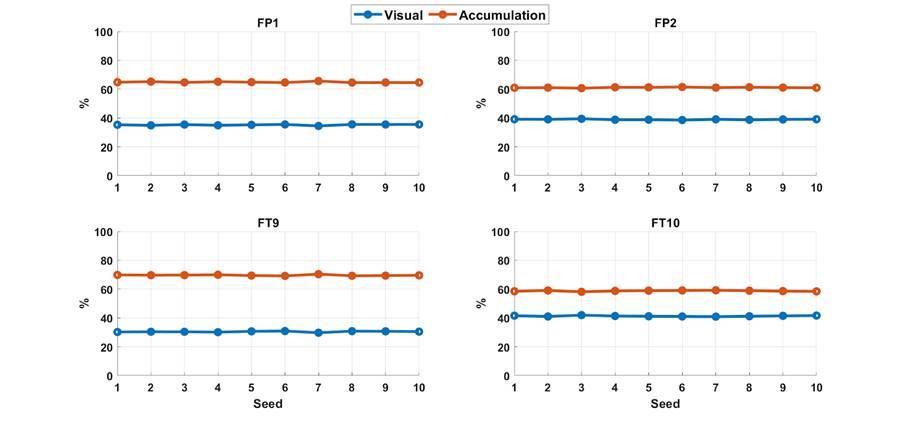


**Figure S7:** For each electrode, the x-axis represents the seeds, while the y-axis indicates the relative percentage contribution of each cluster to the total sum of significant coefficients across both clusters. The orange curve represents the Accumulation cluster, and the blue curve represents the Visual cluster. This visualization highlights both the consistency of cluster contributions across seeds and the relative importance of each cluster in explaining surface ERP signals, with the Accumulation cluster consistently showing a higher contribution with respect to the Visual cluster.

| **Electrode** | **Cluster** | **Brain area** | **N. of predictors** | **x** | **y** | **z** |
| --- | --- | --- | --- | --- | --- | --- |
| **Fp1** | **Visual** | **Fusiform gyrus Pos** | **1** | **38.8** | **-54.5** | **-15.9** |
|  |  | **Inferior temporal gyrus Pos** | **3** | **40.1** | **52.15** | **-5.35** |
|  |  |  |  | **45** | **-1.05** | **-3.2** |
|  |  |  |  | **44.1** | **-53** | **-14.8** |
|  |  | **Middle temporal gyrus Pos** | **3** | **54.3** | **-57.8** | **0.8** |
|  |  |  |  | **45.2** | **-68.6** | **14.1** |
|  |  |  |  | **56.6** | **-64.9** | **6.95** |
|  |  | **White matter** | **2** | **50.3** | **-32.7** | **7.8** |
|  |  |  |  | **27.6** | **-85.2** | **-6.05** |
|  |  | **Posterior cingulate cortex** | **1** | **-12.6** | **-12.6** | **40** |
|  |  | **Inferior parietal cortex** | **2** | **29.2** | **-87.3** | **34.1** |
|  |  |  |  | **32.7** | **-85.3** | **-4.35** |
|  | **Accumulation** | **Superior frontal gyrus** | **9** | **27.75** | **7.75** | **70.6** |
|  |  |  |  | **23.15** | **-5.55** | **64.75** |
|  |  |  |  | **6.45** | **-3.2** | **52.7** |
|  |  |  |  | **10.95** | **-2.45** | **55.2** |
|  |  |  |  | **15.45** | **-1.65** | **57.65** |
|  |  |  |  | **29.6** | **0.25** | **61.95** |
|  |  |  |  | **19.7** | **-0.8** | **65.65** |
|  |  |  |  | **-0.45** | **21.3** | **45.3** |
|  |  |  |  | **-9.1** | **26.8** | **49.35** |
|  |  | **Fusiform gyrus Pos** | **1** | **-47** | **-52.7** | **-19** |
|  |  | **Superior temporal gyrus Pos** | **1** | **-48.7** | **-45** | **8.3** |
|  |  | **Pars orbitalis** | **1** | **32.1** | **39** | **-16.8** |
|  |  | **Fusiform gyrus med** | **2** | **31.1** | **-15** | **-32.3** |
|  |  |  |  | **36.15** | **-15.6** | **-32.4** |
|  |  | **Precentral gyrus dorsal** | **2** | **-14.8** | **-15.1** | **77.6** |
|  |  |  |  | **-25.5** | **-5.65** | **49.55** |
|  |  | **Posterior cingulate cortex** | **1** | **-14.1** | **1.65** | **46.7** |
|  |  | **Caudal middle frontal gyrus** | **2** | **-34.3** | **-1** | **55** |
|  |  |  |  | **-38.5** | **0.9** | **57.85** |
|  |  | **Inferior parietal cortex** | **1** | **44.1** | **-81** | **24.45** |
| **Fp2** | **Visual** | **Inferior temporal gyrus Pos** | **3** | **40.1** | **52.15** | **-5.35** |
|  |  |  |  | **45** | **-1.05** | **-3.2** |
|  |  |  |  | **44.1** | **-53** | **-14.8** |
|  |  | **Middle temporal gyrus Pos** | **3** | **45.2** | **-68.6** | **14.1** |
|  |  |  |  | **40.9** | **-67.4** | **4.85** |
|  |  |  |  | **46.2** | **-66.8** | **5.65** |
|  |  | **White matter** | **2** | **50.3** | **-32.7** | **7.8** |
|  |  |  |  | **27.6** | **-85.2** | **-6.05** |
|  |  | **Superior frontal gyrus** | **1** | **12** | **-5.05** | **58.6** |
|  |  | **Inferior parietal cortex** | **2** | **29.2** | **-86.4** | **31.6** |
|  |  |  |  | **32.7** | **-85.3** | **-4.35** |
|  | **Accumulation** | **Fusiform gyrus Pos** | **3** | **-42.1** | **-52.4** | **-20.5** |
|  |  |  |  | **-47** | **-52.7** | **-19** |
|  |  |  |  | **-38.8** | **-51.3** | **-11.5** |
|  |  | **Superior temporal gyrus Pos** | **1** | **-48.7** | **-45** | **8.3** |
|  |  | **Superior frontal gyrus** | **8** | **23.15** | **-5.55** | **64.75** |
|  |  |  |  | **6.45** | **-3.2** | **52.7** |
|  |  |  |  | **10.95** | **-2.45** | **55.2** |
|  |  |  |  | **15.45** | **-1.65** | **57.65** |
|  |  |  |  | **19.7** | **-0.8** | **65.65** |
|  |  |  |  | **-7.15** | **4.5** | **64.4** |
|  |  |  |  | **-0.45** | **21.3** | **45.3** |
|  |  |  |  | **-4.8** | **23.95** | **46.95** |
|  |  | **Supramarginal gyrus** | **1** | **48.05** | **-38.5** | **51.8** |
|  |  | **Pars orbitalis** | **1** | **32.1** | **39** | **-16.8** |
|  |  | **Fusiform gyrus med** | **1** | **36.15** | **-15.6** | **-32.4** |
|  |  | **Precentral gyrus dorsal** | **2** | **-14.8** | **-15.1** | **77.6** |
|  |  |  |  | **-25.5** | **-5.65** | **49.55** |
|  |  | **Caudal middle frontal gyrus** | **2** | **-34.3** | **-1** | **55** |
|  |  |  |  | **-38.5** | **0.9** | **57.85** |
|  |  | **Inferior parietal cortex** | **1** | **44.1** | **-81** | **24.45** |
| **FT9** | **Visual** | **Inferior temporal gyrus Pos** | **2** | **40.1** | **52.15** | **-5.35** |
|  |  |  |  | **45** | **-1.05** | **-3.2** |
|  |  | **Middle temporal gyrus Pos** | **3** | **54.3** | **-57.8** | **0.8** |
|  |  |  |  | **45.2** | **-68.6** | **14.1** |
|  |  |  |  | **56.6** | **-64.9** | **6.95** |
|  |  | **Posterior cingulate cortex** | **1** | **-12.6** | **-12.6** | **40** |
|  |  | **Fusiform gyrus Pos** | **1** | **38.8** | **-54.5** | **-15.9** |
|  |  | **Inferior parietal cortex** | **2** | **29.2** | **-87.3** | **34.1** |
|  |  |  |  | **32.7** | **-85.3** | **-4.35** |
|  |  | **White matter** | **2** | **35.6** | **-68.5** | **3.55** |
|  |  |  |  | **27.6** | **-85.2** | **-6.05** |
|  | **Accumulation** | **Fusiform gyrus Pos** | **1** | **-47** | **-52.7** | **-19** |
|  |  | **Superior temporal gyrus Pos** | **1** | **-48.7** | **-45** | **8.3** |
|  |  | **Superior frontal gyrus** | **9** | **18.75** | **11.2** | **71.65** |
|  |  |  |  | **27.75** | **7.75** | **70.6** |
|  |  |  |  | **23.15** | **-5.55** | **64.75** |
|  |  |  |  | **6.45** | **-3.2** | **52.7** |
|  |  |  |  | **10.95** | **-2.45** | **55.2** |
|  |  |  |  | **15.45** | **-1.65** | **57.65** |
|  |  |  |  | **29.6** | **0.25** | **61.95** |
|  |  |  |  | **19.7** | **-0.8** | **65.65** |
|  |  |  |  | **-0.45** | **21.3** | **45.3** |
|  |  | **Pars orbitalis** | **1** | **32.1** | **39** | **-16.8** |
|  |  | **Fusiform gyrus med** | **2** | **31.1** | **-15** | **-32.3** |
|  |  |  |  | **36.15** | **-15.6** | **-32.4** |
|  |  | **Precentral gyrus dorsal** | **3** | **-14.8** | **-15.1** | **77.6** |
|  |  |  |  | **-20.9** | **-8** | **46.05** |
|  |  |  |  | **-25.5** | **-5.65** | **49.55** |
|  |  | **Posterior cingulate cortex** | **1** | **-14.1** | **1.65** | **46.7** |
|  |  | **Caudal middle frontal gyrus** | **2** | **-34.3** | **-1** | **55** |
|  |  |  |  | **-38.5** | **0.9** | **57.85** |
| **FT10** | **Visual** | **Inferior temporal gyrus Pos** | **3** | **40.1** | **52.15** | **-5.35** |
|  |  |  |  | **45** | **-1.05** | **-3.2** |
|  |  |  |  | **44.1** | **-53** | **-14.8** |
|  |  |  |  |  |  |  |
|  |  | **White matter** | **2** | **50.3** | **-32.7** | **7.8** |
|  |  |  |  | **27.6** | **-85.2** | **-6.05** |
|  |  | **Superior frontal gyrus** | **1** | **12** | **-5.05** | **58.6** |
|  |  | **Fusiform gyrus med** | **1** | **32.4** | **-47.3** | **-14.1** |
|  |  | **Fusiform gyrus Pos** | **1** | **38.8** | **-54.5** | **-15.9** |
|  |  | **Inferior parietal cortex** | **3** | **29.2** | **-86.4** | **31.6** |
|  |  |  |  | **29.2** | **-87.3** | **34.1** |
|  |  |  |  | **32.7** | **-85.3** | **-4.35** |
|  |  | **Middle temporal gyrus Pos** | **1** | **45.2** | **-68.6** | **14.1** |
|  | **Accumulation** | **Fusiform gyrus Pos** | **3** | **-42.1** | **-52.4** | **-20.5** |
|  |  |  |  | **-47** | **-52.7** | **-19** |
|  |  |  |  | **-38.8** | **-51.3** | **-11.5** |
|  |  | **Superior temporal gyrus Pos** | **1** | **-48.7** | **-45** | **8.3** |
|  |  | **White matter** | **1** | **-52.1** | **-43.9** | **16.75** |
|  |  | **Superior frontal gyrus** | **7** | **23.15** | **-5.55** | **64.75** |
|  |  |  |  | **6.45** | **-3.2** | **52.7** |
|  |  |  |  | **15.45** | **-1.65** | **57.65** |
|  |  |  |  | **19.7** | **-0.8** | **65.65** |
|  |  |  |  | **-17.2** | **17.35** | **67.05** |
|  |  |  |  | **-0.45**  **-9.1** | **21.3**  **26.8** | **45.3**  **49.35** |
|  |  | **Pars orbitalis** | **1** | **32.1** | **39** | **-16.8** |
|  |  | **Fusiform gyrus med** | **1** | **36.15** | **-15.6** | **-32.4** |
|  |  | **Precentral gyrus dorsal** | **3** | **-14.8** | **-15.1** | **77.6** |
|  |  |  |  | **-25.5** | **-5.65** | **49.55** |
|  |  |  |  | **-30.2** | **-3** | **52.2** |
|  |  | **Caudal middle frontal gyrus** | **2** | **-34.3** | **-1** | **55** |
|  |  |  |  | **-38.5** | **0.9** | **57.85** |
|  |  | **Inferior parietal cortex** | **1** | **44.1** | **-81** | **24.45** |

**Table S1:** Anatomical localization of reliable predictors for each cluster and surface electrode.

**References**

MathWorks (2024a) fitlme. Available at: <https://fr.mathworks.com/help/stats/fitlme.html> (Accessed: 1 December 2024).

MathWorks (2024b) Prepare Data for Linear Mixed-Effects Models. Available at: <https://fr.mathworks.com/help/stats/prepare-data-for-linear-mixed-effects-models.html> (Accessed: 1 December 2024).

MathWorks (2024c) ridge. Available at: <https://fr.mathworks.com/help/stats/ridge.html> (Accessed: 1 December 2024).

Özkale, M.R. and Altuner, H. (2023) 'Bootstrap confidence interval of ridge regression in linear regression model: A comparative study via a simulation study', Communications in Statistics - Theory and Methods, 52(20), pp. 7405–7441
